# Transcriptional Profiling of Rice Treated with MoHrip1 Reveal the Function of Protein Elicitor in Enhancement of Disease Resistance and Plant Growth

**DOI:** 10.3389/fpls.2016.01818

**Published:** 2016-12-01

**Authors:** Shun Lv, Zhenzhen Wang, Xiufen Yang, Lihua Guo, Dewen Qiu, Hongmei Zeng

**Affiliations:** State Key Laboratory for Biology of Plant Diseases and Insect Pests, Institute of Plant Protection, Chinese Academy of Agricultural SciencesBeijing, China

**Keywords:** elicitor, MoHrip1, DGE (digital gene expression profiling) sequencing, disease-resistance, signaling pathways

## Abstract

MoHrip1 is a protein elicitor isolated from *Magnaporthe oryzae* and was found to induce blast-resistance in rice. To investigate the comprehensive functions of MoHrip1, next-generation sequencing (NGS)-based digital gene expression (DGE) profiling was performed to collect the transcriptional data of differentially expressed genes (DEGs) induced by MoHrip1. A total of 308 genes were identified with differential expression, and 80 genes were predicted to be induced specifically by MoHrip1. Among these 308 genes, a series of genes associated with the salicylic acid (SA) pathway, phytoalexin, transcription factors, and pathogen-related proteins were identified. Both the SA signaling pathway and the gibberellin (GA) pathway were activated, while the jasmonic acid (JA) signaling pathway was repressed. The contents of endogenous SA and GA and the morphological characteristics of the rice after treatment were measured to provide evidence supporting the predictions made based on the DGE data. The 80 genes mentioned above might be candidate genes for studying interactions with MoHrip1. The transcriptional data provided global effect information in rice induced by MoHrip1, and all the results demonstrated that MoHrip1 could induce pathogen resistance and promote plant growth by regulating the contents of SA and GA directly or indirectly.

## Introduction

Rice blast, a major fungal disease caused by *Magnaporthe oryzae*, seriously affects rice production worldwide (Ribot et al., 2008), leading to yield losses of 50–70% (Bagnaresi et al., 2012). As rice is the world's second most important human food crop (FAOSTAT, 2014), it is necessary to develop methods to protect it from pathogenic diseases and to increase its production. Increasing research on the molecular events governing the interactions between rice and *M. oryzae* have contributed to methods for improving disease resistance (Chen and Ronald, 2011).

The elicitors include molecules isolated from various organisms, including viruses, oomycetes, bacteria, and fungi. Some elicitors have been used to improve the pathogen resistance of plants (Bent and Mackey, 2007). Such elicitor molecules include oligosaccharides, lipids, peptides, glycoproteins, and proteins (Nürnberger, 1999; De Wit et al., 2009; Ellis et al., 2009). A range of elicitors was isolated from *M. oryzae*, including sphingolipids, proteins, and glycoproteins. These elicitors induce resistance responses and hypersensitive cell death in rice, while inducing systemic acquired resistance (SAR) in plants (Koga et al., 1998; Qiu et al., 2009; Peng et al., 2011). However, these elicitor-activity compounds were not secretory proteins. During the whole genome sequencing and research on the *M. oryzae* proteome, several secreted proteins were identified and characterized, such as MC69 (Saitoh et al., 2012), SLP1 (Mentlak et al., 2012), Avr-effectors (PWL1, PWL2, AvrPi-ta, AvrPiz-t, Avr-Pia, AvrPii, Avr-Pik/km/kp, Avr1-CO39, ACE1) (Liu et al., 2013), and MoCDIP1-5 (Chen et al., 2013). MoHrip1 is a secreted protein elicitor isolated from *M. oryzae* and can enhance the defense of rice seedlings against infection by *M. oryzae* (Chen et al., 2012).

Next-generation sequencing (NGS) technologies are rapidly evolving and are changing biology research (Metzker, 2010). Digital gene expression (DGE) is a relatively new approach based on NGS technology for the study of the transcriptome, and this method has advantages in studying genes that are expressed differentially (Tao et al., 2012). The sequencing of the Rice cv. Nipponbare (*Oryza sativa* spp. japonica) genome has been implemented and published (Sasaki and Burr, 2000; Kawahara et al., 2013), making sequencing-based transcriptome studies (i.e., DGE) more accessible. To our knowledge, there have been some RNA-seq studies focusing on rice resistance genes and the interactions between pathogens and rice (Bagnaresi et al., 2012). Furthermore, several studies aiming to identify differences in the gene expression in rice suspension cells treated with elicitors have been completed (Kim et al., 2000). However, no NGS-based transcriptome studies have been conducted focusing on genes induced by the mutual effects of intact rice and pathogen-derived elicitors.

In this research, we employed DGE-based RNA-seq to study the gene expression in rice induced by MoHrip1. Comparisons between the treatment and controls allowed us to identify genes sensitive to the elicitors. Many differentially expressed genes (DEGs) related to the defense response were identified, along with genes associated with plant growth. Even some specific genes that were not expressed in untreated rice but that were up-regulated in rice after treatment were identified. Our results may widen the functional scope of the elicitor in inducing resistance and may help identify genes that interact with the elicitor.

## Methods

### Plant material and elicitor preparation

Rice cv. Nipponbare (*O. sativa* spp. japonica) was used in this study. For RNA-seq, the rice seeds were sterilized by immersion in 2% sodium hypochlorite for 30 min. After they were washed with deionized water 7–8 times, the seeds were germinated on 1/2 Murashige and Skoog (MS) medium for 5 days and transferred to nutritional soil (Park et al., 2012). Rice plants were cultured in a growth chamber at 30°C in light and 25°C in dark, with a 12-h light/dark photoperiod. We treated the rice seedlings by spraying with either elicitor (30 μM) or Tris-Cl (25 mM) as a negative control at the three-leaf stage. Both the treated rice and the negative control were sampled in two replicates at 0, 24, 48, 72 h after treatment (hat), separately. Samples for RNA-seq were collected and frozen in liquid nitrogen and stored at −80°C until use.

The MoHrip1 used in this study was expressed and purified as previously described (Chen et al., 2012; Zhang et al., 2013). The recombinant expression vector containing the MoHrip1 gene was transformed into *E. coli* strain transetta (DE3) competent cells (TransGen Biotech, Beijing, China) to express the elicitor. The bacteria were first grown to achieve an OD_600_ of 0.8 in Luria Bertani (LB) medium at 37°C. Then, the protein was induced upon the addition of 0.1 mM isopropyl β-D-1-thiogalactopyranoside (Sigma, St. Louis, MO, USA) at 16°C. Twelve hours after induction, the cells were collected by centrifugation and resuspension. After ultrasonication and centrifugation, the supernatant containing the recombinant protein was obtained. The protein purification mainly consisted of two procedures: affinity chromatography with a His-Trap HP column (GE Healthcare, Waukesha, WI, USA) and ion-exchange chromatography with a Mono Q column (GE Healthcare, Waukesha, WI, USA). After concentration, the purified protein was detected by SDS-PAGE and was quantified using the BCA™ Protein Assay Kit (Pierce, Rockford, IL, USA).

### DGE library preparation and sequencing

A total of 14 samples (two replicates of non-treated rice and two replicates of treated and control rice collected at three time points) were prepared for RNA extraction. The total RNA was isolated using an RNAprep Plant Kit (TIANGEN Biotech, Beijing, China) according to the manufacturer's protocol. RNA purity was checked using a NanoPhotometer® spectrophotometer (IMPLEN, CA, USA). RNA integrity was confirmed using an RNA Nano 6000 Assay Kit for the Agilent Bioanalyzer 2100 system (Agilent Technologies, CA, USA) with a minimum RNA integrated number (RIN) value of 7.

A total of 3 μg of RNA per sample was used as the input material for the RNA sample preparations. Sequencing libraries were generated using the NEBNext® Ultra™ RNA Library Prep Kit for Illumina® (NEB, USA) following the manufacturer's recommendations. In brief, mRNA was purified from the total RNA using oligo (dT) magnetic beads. After fragmentation with divalent cations in NEBNext First Strand Synthesis Reaction Buffer (5X), first strand cDNA was synthesized using random hexamer primers and M-MuLV Reverse Transcriptase (RNase H^−^). Then, second strand cDNA synthesis was performed using DNA Polymerase I and RNase H. To select cDNA fragments 150–200 bp in length, the library fragments were purified using the AMPure XP system (Beckman Coulter, Beverly, USA). PCR was then performed with Phusion High-Fidelity DNA polymerase, Universal PCR primers and an Index (X) Primer. Finally, the PCR products were purified (AMPure XP system), and the library quality was assessed on an Agilent Bioanalyzer 2100 system.

The clustering of the samples was performed on a cBot Cluster Generation System using a TruSeq PE Cluster Kit v3-cBot-HS (Illumina) according to the manufacturer's instructions. After cluster generation, the library was sequenced on an Illumina HiSeq 2000 platform, and 100-bp paired-end reads were generated.

### RNA-seq read mapping and DE gene clustering

The sequence data sets are available at the NCBI Short Read Archive (SRA) under the accession number SRA325858. Raw data had adaptor fragments and a few low-quality sequences, along with several types of impurities. Clean data were obtained by removing low-quality reads containing adapters or poly-N sequences from the raw data. All downstream analyses were based on clean, high-quality data.

The reference genome and gene model annotation files were downloaded from the genome website directly. The reference genome index was built using Bowtie v2.2.3 (Langmead et al., 2009), and paired-end clean reads were aligned to the reference genome using TopHat v2.0.12 (Trapnell et al., 2009). TopHat was selected as the mapping tool, as it has the advantage of being able to generate a database of splice junctions based on the gene model annotation file and, thus, to produce better mapping results than other non-splice mapping tools. HTSeq v0.6.1 (Anders, 2010) was used to count the read numbers mapped to each gene. The reads per kilobase of transcript per million mapped reads (RPKM) of each gene was calculated based on the length of the gene and the read counts mapped to that gene (Mortazavi et al., 2008).

### Analysis of differentially expressed genes

The DESeq R package (1.10.1) (Wang et al., 2010) was introduced to perform differential expression analysis of two conditions (two biological replicates per condition). DESeq (Anders and Huber, 2010) provides statistical methods for identifying differential expression in DGE data using a model based on the negative binomial distribution. The resulting *P*-values were adjusted using the Benjamini and Hochberg's approach for controlling the false discovery rate (FDR) (Benjamini and Hochberg, 1995). Genes with a FDR adjusted *P* < 0.05, as found by DESeq, were defined as being significant differentially expressed.

Gene Ontology (GO) enrichment analysis of the DEGs was implemented in the GOseq (Young et al., 2010) R package, in which the gene length bias was corrected. GO terms with corrected *P* < 0.05 were considered significantly enriched in the DEGs. KEGG (Kyoto Encyclopedia of Genes and Genomes) (Kanehisa et al., 2008) is a database resource for understanding the high-level functions and utilities of a biological system. KEGG has abundant molecular-level information, especially in the format of large-scale molecular datasets generated by genome sequencing and other high-throughput experimental technologies. KOBAS (Mao et al., 2005) software was used to test the statistical enrichment of the DEGs in the KEGG pathway.

### Real-time quantitative RT-PCR assay

For each condition, three independent RNA samples were used to validate gene expression level by performing quantitative real-time PCR. The cDNAs used as the template of Quantitative RT-PCR were produced using the TransScript All-in-One First-Strand cDNA Synthesis SuperMix for qPCR (TransGen Biotech, Beijing, China), and the concentrations of the mRNAs were adjusted to be the same. Quantitative RT-PCR was performed using SuperReal PreMix Plus (TIANGEN Biotech, Beijing, China). Each real-time RT-PCR reaction (20 μL) included 25 ng cDNA, 0.3 μM of each primer, and 1 × SYBR Green PreMix. All reactions were performed in three technical replicates on a Bio-rad CFX96 Real-Time PCR Detection System (Bio-Rad, CA, USA) under the following conditions: 95°C for 15 min and 40 cycles of 95°C for 10 s, 55°C for 30 s, and 72°C for 32 s to determine the fluorescence level and to calculate cycle threshold (Ct) values, followed by increments of 0.5°C for 5 s along a gradient from 65°C to 95°C. Melting curves were obtained to ensure primer specificity. Gene relative expression level was obtained by comparing with non-treated rice after normalization with reference gene using the 2^−ΔΔCt^ approach. Osactin was used as the reference gene, and the gene IDs and primer sequences are listed in Table S1. Data were statistically validated by a correlation test using the Pearson's method.

### Determination of the endogenous levels of salicylic acid (SA)

For the SA content assay, the rice cultivation method was the same as was used for the RNA-seq. Treated and control rice samples were collected in three replicates at 0, 12, 24, 36, 48, 60, 72 h after treatment (hat). Leaf tissue treated with MoHrip1 or buffer was collected, weighed and frozen in liquid nitrogen. For each sample, 0.1 g of the frozen tissue was extracted and quantitated for free SA, as described previously (Bowling et al., 1994). In brief, the tissue was ground into powder and homogenized in 1 mL of methanol-H_2_O-acetic acid (80:19:1). After extraction overnight at 4°C and centrifugation, the supernatant was re-extracted with the solution described previously. After the addition of 1 mL chloroform and further centrifugation, the organic phase containing the free SA was dried in a speed vacuum with heat (~40°C). The residue was resuspended in 0.5 mL of methanol, filtered and analyzed by Ultra Performance Liquid Chromatography (UPLC). UPLC was performed on an ACQUITY UPLC@BEHC_18_ column (50 mm × 2.1 mm, 1.7 μm) run at 40°C with a flow rate of 0.4 mL min^−1^. The analytes were eluted from the column with a mixed solvent of water with 0.1% acetic acid (solvent A) and methanol with 0.1% acetic acid (solvent B) using a linear gradient mode (Matsuura et al., 2009). The ratio of A and B was 90:10 from 0 s to 3 min, and this ratio changed linearly from 90:10 to 10:90 between 3 and 4 min. The ratio of 90:10 was finally maintained from 4 to 7 min. The authenticity of the SA from rice leaf extract was verified based on the retention times and spectral properties, which matched perfectly to those of commercial SA standards.

### Growth promotion assay and determination of endogenous levels of GA

For the GA assay, the rice seeds were sterilized by immersion in 75% ethanol for 5 min. After they were washed with sterile water, seeds were immersed in different concentrations of elicitor dilution (2.5, 5, 10, and 20 μg/mL) or H_2_O as a negative control for 8 h. Then, the germinated seeds were transferred to liquid MS medium for cultivation. The leaf tissue was collected at 9, 11, and 13 days after the rice seedlings were transferred to liquid MS medium with three replicates. For the growth promotion assay, the lengths of the seedlings and roots were measured, as were the weights of the whole plants. To assess the endogenous GA levels, the leaf tissue was ground into powder in liquid nitrogen after the weight measurement and addition of phosphate buffer (pH 7.4, 0.01 mol/L), with a ratio of 1:9. The supernatant was collected after centrifugation. Then, the GA level was determined using a Plant Gibberellic Acid ELISA Kit (FUYING Biotech, Shanghai, China) following the solid-phase enzyme immunoassay method (Atzorn and Weiler, 1983). In brief, the supernatant was added to a microtiter plate coated well with purified plant GA antibody and was then incubated at 37°C. The chromogen solution was then added following the addition of horseradish peroxidase (HRP)-conjugate reagent and incubated at 37°C. After adding the stop solution, the absorbance was obtained at 450 nm and was matched with the GA standard curve to calculate the endogenous GA levels of the leaf tissues.

### Bioassay for MoHrip1-induced disease resistance in rice

For the disease resistance assay, the rice seeds were divided into three groups. Each group contains 25 rice seeds with three replicates. The MM group indicated that the rice seeds were immersed in MoHrip1 (10 μg/mL) as the GA assay then sprayed with MoHrip1 (30 μM) at three-leaf stage as assay for RNA-seq mentioned above. The M group indicated that the rice seeds were immersed in MoHrip1 (10 μg/mL) then sprayed with Tris-Cl (25 mM) at three-leaf stage. The CK group indicated that the rice seeds were immersed in H_2_O then sprayed with Tris-Cl (25 mM) at three-leaf stage as a negative control. After 3 days of incubation, three groups of rice plants were sprayed with an aqueous suspension of 1 × 106 *M. oryzae* (KJ201) spores per milliliter containing 0.05% (v/v) Tween 20. The inoculated rice plants were maintained at 26°C and 100% relative humidity in a dark chamber for 24 h. Then we modified the growth chamber with 70–80% relative humidity under a 14-h-light/10-h-dark photoperiod (Chen et al., 2012). Leaf blast symptoms were investigated at 7 days post-inoculation when typical lesions appeared on the leaves of negative control rice plants. The disease indices of plants inoculated with rice blast were compared. Each seedling was researched and rated on a scale of 0–9 (0 = resistant and 9 = susceptible) according to the international specification for rice blast disease.

## Results

### DGE sequencing of the MoHrip1-treated rice

A previous study confirmed that rice treated with MoHrip1 show enhanced resistance to *M. oryzae* (Chen et al., 2012). To obtain the global gene expression profile differences of rice treated with MoHrip1 compared to those treated with buffer (Tris-Cl) as a negative control, 24, 48, and 72 hat and non-treated rice samples were prepared and sequenced on the Illumina sequencing platform. A total of 14 DGE libraries were constructed to identify differences in the gene expression levels. The 14 libraries included non-treated rice samples (designated as NoT0), rice samples of MoHrip1 and buffer at 24 hat (designated as MoT1 and BuT1), rice samples of MoHrip1 and buffer at 48 hat (designated as MoT2 and BuT2), and rice samples of MoHrip1 and buffer at 72 hat (designated as MoT3 and BuT3), with two replicates each. After removing reads containing adapters, reads containing poly-N sequences, and low-quality reads, the total clean reads per library ranged from 10.3 to 14.8 million. The total reads mapped to the Rice cv. Nipponbare genome according to the TopHat analysis ranged from 9.5 to 14.4 million (Table 1). RPKM was introduced to calculate the expression level of each gene. The value of “RPKM ≥ 1 or < 1” was used as the threshold to identify whether a gene was expressed or not (Table 2).

**Table 1:** **Statistics of DGE sequencing of 14 libraries of non-treated rice (NoT0) and treated rice at 24, 48, and 72 h after treatment with MoHrip1 or buffer (MoT1, MoT2, MoT3, and BuT1, BuT2, BuT3)**.

		**NoT0_1**	**NoT0_2**	**MoT1_1**	**MoT1_2**	**BuT1_1**	**BuT1_2**	**MoT2_1**	**MoT2_2**	**BuT2_1**	**BuT2_2**	**MoT3_1**	**MoT3_2**	**BuT3_1**	**BuT3_2**
Raw reads	Number	13805789	13332944	13647457	12474959	13119481	13594058	14995442	14782707	11517416	11148463	11414761	10438482	14720906	12792358
Clean reads(total reads)	Number	13741369	13283801	13584649	12422320	13024454	13463535	14827415	14599410	11416104	11044016	11310928	10337594	14604918	12639088
Total mapped	Number	13286896	12849471	13137715	12016674	12517076	12942714	14446290	14181585	10685155	10281327	10544742	9566225	14165590	12249696
	% of number	96.69	96.73	96.71	96.73	96.10	96.13	97.43	97.14	93.60	93.09	93.23	92.54	96.99	96.92
Multiple mapped	number	388616	371806	407752	372485	361022	389364	460567	435915	343212	326238	338155	328584	446230	356151
	% of number	2.83	2.80	3.00	3.00	2.77	2.89	3.11	2.99	3.01	2.95	2.99	3.18	3.06	2.82
Uniquely mapped	Number	12898280	12477665	12729963	11644189	12156054	12553350	13985723	13745670	10341943	9955089	10206587	9237641	13719360	11893545
	% of number	93.86	93.93	93.71	93.74	93.33	93.24	94.32	94.15	90.59	90.14	90.24	89.36	93.94	94.10

*Each condition represents two replicates*.

**Table 2:** **Statistics for the gene numbers in the different RPKM intervals**.

**RPKM Interval**	**NoT0_1**	**NoT0_2**	**MoT1_1**	**MoT1_2**	**BuT1_1**	**BuT1_2**	**MoT2_1**	**MoT2_2**	**BuT2_1**	**BuT2_2**	**MoT3_1**	**MoT3_2**	**BuT3_1**	**BuT3_2**
0–1	19295	19077	17614	17458	17729	18123	17539	17347	18151	18425	17894	18777	18418	19149
	(54.08%)	(53.47%)	(49.37%)	(48.93%)	(49.69%)	(50.79%)	(49.16%)	(48.62%)	(50.87%)	(51.64%)	(50.15%)	(52.63%)	(51.62%)	(53.67%)
1–3	4135	4013	4075	3926	4066	4106	3859	3908	3899	3950	4040	4113	3942	4115
	(11.59%)	(11.25%)	(11.42%)	(11.00%)	(11.40%)	(11.51%)	(10.82%)	(10.95%)	(10.93%)	(11.07%)	(11.32%)	(11.53%)	(11.05%)	(11.53%)
3–15	7449	7502	7795	7699	7659	7712	7648	7757	7605	7562	7650	7543	7480	7432
	(20.88%)	(21.03%)	(21.85%)	(21.58%)	(21.47%)	(21.61%)	(21.44%)	(21.74%)	(21.32%)	(21.19%)	(21.44%)	(21.14%)	(20.96%)	(20.83%)
15–60	3484	3711	4557	4848	4551	4238	4889	4893	4428	4207	4488	3834	4282	3614
	(9.76%)	(10.40%)	(12.77%)	(13.59%)	(12.76%)	(11.88%)	(13.70%)	(13.71%)	(12.41%)	(11.79%)	(12.58%)	(10.75%)	(12.00%)	(10.13%)
>60	1316	1376	1638	1748	1674	1500	1744	1774	1596	1535	1607	1412	1557	1369
	(3.69%)	(3.86%)	(4.59%)	(4.90%)	(4.69%)	(4.20%)	(4.89%)	(4.97%)	(4.47%)	(4.30%)	(4.50%)	(3.96%)	(4.36%)	(3.84%)

A saturation curve was constructed to determine whether the amount of data fulfilled the requirement of quantifying the gene expression level. The higher the gene expression level, the easier it was to accurately quantify the gene (Figure S1). Biological replicates were necessary for high-throughput sequencing to obtain reliable analysis results (Hansen et al., 2011). Our experiments showed extremely high levels of correlation for each pair of biological replicates [in all cases examined, the Pearson correlation coefficient (PCC) >0.97; Figure S2], indicating that our sampling, assay, and analysis methods were steady and robust.

GO enrichment analysis was performed to classify the gene functions of the detected DEGs. Based on the sequencing data, the genes detected differentially in the treated rice after 1 day were enriched in two domains: biological process and molecular function. The cardinal enriched terms were metabolic and oxidation-reduction processes in the biological process domain and oxidoreductase lyase activity in molecular function. Genes detected differentially in treated rice after 2 days were enriched in only one domain: the biological process domain, and the cardinal term was metabolic process. In contrast, the genes detected differentially in the treated rice after 3 days were not found to be enriched (Figure S3).

To characterize the complex biological behaviors for the transcriptome, the KEGG database was used to analyze the pathway annotations for the DEGs. In the three rice groups being compared between the treatment and control conditions, only genes in the rice treated for 1 day showed significant pathway enrichment. The pathways with the most representation were the phenylalanine, tyrosine, tryptophan, diterpenoid, phenylpropanoid, amino acid, and secondary metabolite biosynthesis pathways and the plant-pathogen interaction pathways (Figure S4; Table S2). These annotations provided clues for investigating specific processes, especially those involved in the biosynthesis of secondary metabolites and plant-pathogen interactions.

**Figure 1:**
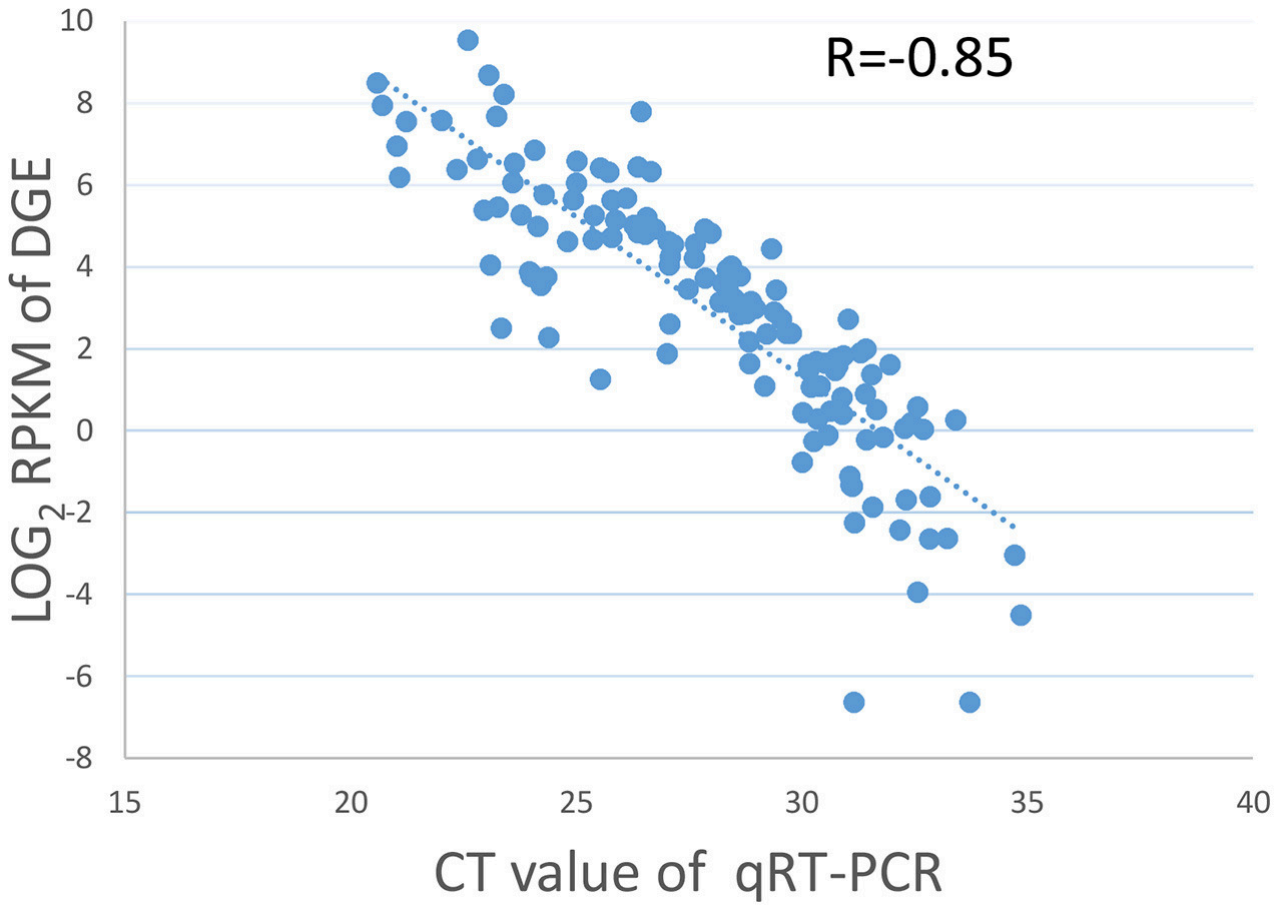
**Validation of DGE data by qRT-PCR**. Scatter plots indicate the Ct results of the qRT-PCR and the log_2_ RPKM values of DGE for 133 data points from 19 genes in 7 different samples with a Pearson correlation coefficient of *R* = −0.85.

Nineteen genes with a range of expression levels were selected for the validation of the gene expression levels estimated by RPKM using quantitative RT-PCR (qRT-PCR). The expression levels of the 19 genes determined by the qRT-PCR analysis using 7 different RNA samples generated 133 data points. The data for the cycle threshold (Ct) value and the log_2_ RPKM value of the selected genes are given in Table S3. The CT value of the qRT-PCR results correlated well with the log_2_ RPKM values of the DGE, with a PCC of *R* = −0.85 (Figure 1), indicating that the gene expression levels determined by DGE analysis were positively correlated with those determined by qRT-PCR.

### Differential gene expression analysis

To identify the genes induced by treatment with MoHrip1 and to assess gene expression pattern changes, we next evaluated the DEGs at all-time points. The final RPKM of one gene with replicates in the same condition was the average value for all replicate data. In general, the absolute value of “|log_2_(FoldChange)| > 1” and the “*q* < 0.005” were used as the threshold to identify the DEGs in a sequencing experiment without replicates. Now that DESeq was used to eliminate biological deviation in the study with replicates, we set padj < 0.05 as filtering standard for genes that were expressed differentially.

The generated libraries formed 9 volcano plots and 3 Venn diagrams based on 9 DEG comparisons (MoT1 vs. NoT0, MoT2 vs. NoT0, MoT3 vs. NoT0, BuT1 vs. NoT0, BuT2 vs. NoT0, BuT3 vs. NoT0, MoT1 vs. BuT1, MoT2 vs. BuT2, and MoT3 vs. BuT3). Based on the volcano plot, 214 DEGs were obtained at 24 hat, including 163 up-regulated genes and 51 down-regulated genes. Furthermore, 103 DEGs were obtained at 48 hat, including 78 up-regulated genes and 25 down-regulated genes. Finally, 21 DEGs were obtained at 72 hat, including 14 up-regulated genes and 7 down-regulated genes (Figure S5). A Venn diagram was used to obtain the overlapping DEGs in the comparison group to see which DGEs were induced transiently and which DGEs were induced over a relatively long period of time. In this experiment, 28 genes were induced at both 24 and 48 h, 1 gene was induced at both 24 and 72 h, and 1 gene was induced at both 48 and 72 h, indicating that these genes may play important roles in induced resistance after treatment with MoHrip1 (Figure S6A). It can also be concluded through the volcano plot and Venn diagram that both the treatment and the control conditions could induce gene expression differentially compared with the non-treated condition, while the treated samples induced more DGEs relative to the non-treated condition than the control samples (Figures S6B,C, S7).

### Differences in gene expression patterns as assessed by clustering analysis

**Figure 2:**
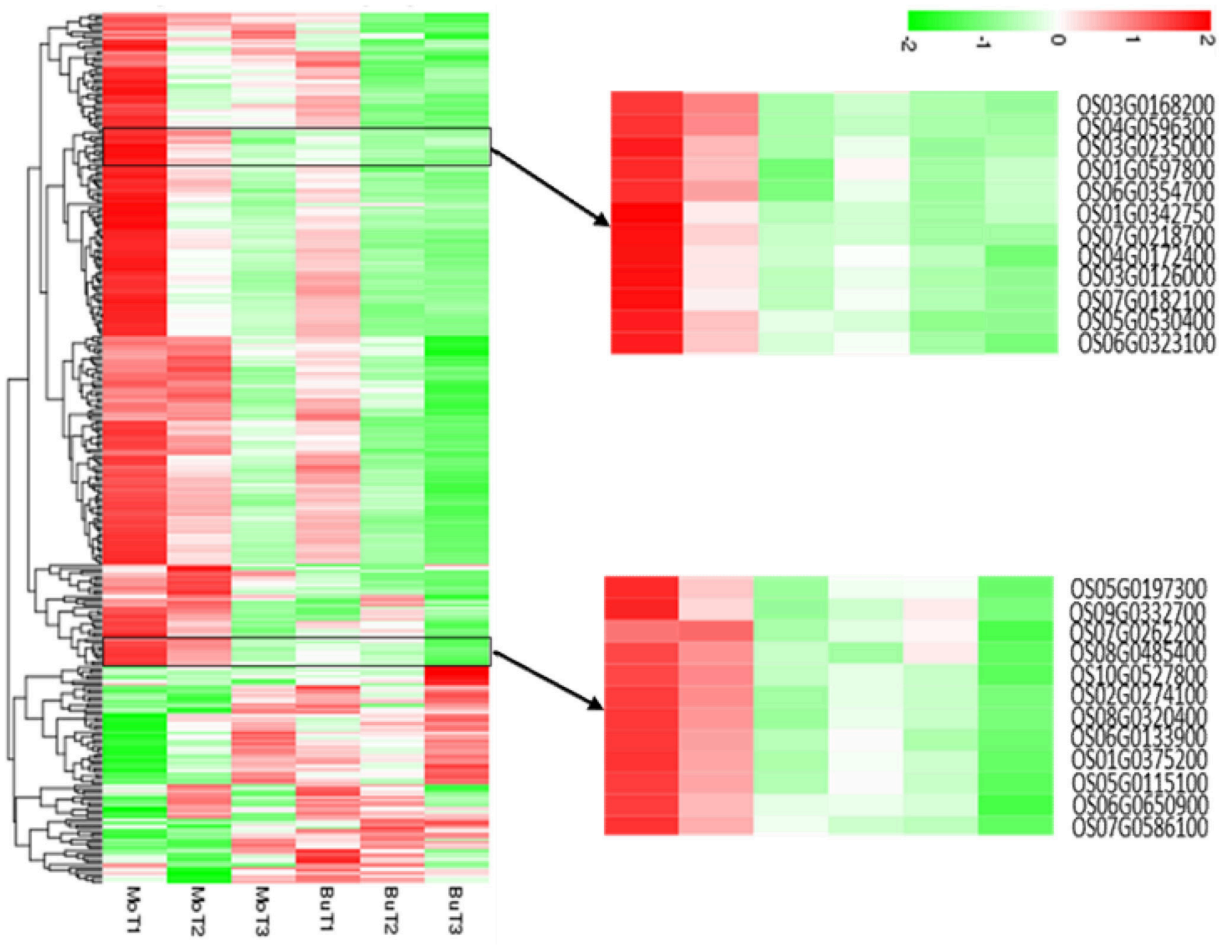
**Hierarchical clustering of the differentially expressed genes**. The heat map shows the gene expression clusters and sample clusters. Each line indicates data from a single gene. The color bar represents the log_10_(RPKM) of each gene, ranging from green (−2) to red (2). Two detailed gene clusters show the genes that are up-regulated in the MoHrip1-treated samples after 24 and 48 h and down-regulated in the buffer-treated samples after 24, 48, and 72 h.

A total of 308 DEGs (|log_2_(FoldChange)| > 1, *q* < 0.005) were identified when rice samples treated with MoHrip1 or buffer after 24, 48, 72 h were compared with non-treated rice samples. All genes were used to generate clusters using the clustering affinity search technique (CAST). We identified two representative hierarchical clusters, consisting of a total of 24 genes, showing interesting regulation patterns at 24 and 48 hat (Figure 2). In the MoHrip1-treated group, these 24 genes were induced at 24 and 48 hat; in the buffer-treated group, these 24 genes were suppressed at all-time intervals that we analyzed (24, 48, 72 hat). These genes included peroxidase N (prxRPN), the ABC transporter (PDR20), and the heat stress transcription factor (Spl7). Two genes were specifically associated with the shikimate pathway (DHQDT/SDH, EPSPS) and three genes were specifically associated with the tryptophan pathway (IGPS, PAT, TS; Table S4) (Maeda and Dudareva, 2012). These shikimate and tryptophan pathway-associated genes were also included in the KEGG enrichment (data not shown). These results suggest that MoHrip1 can induce genes in several ways in rice, while the buffer has no such effect.

### DGE analysis reveals the molecular events induced by MoHrip1 in rice

Previous studies have identified genes and pathways that participate in or are related to the resistance of blast. In our DGE data, we examined how these genes were expressed in rice after treatment with MoHrip1. This research aimed to identify genes or pathways critical for blast-resistance in rice, especially those induced by MoHrip1.

### Pathogenesis-related proteins

**Figure 3:**
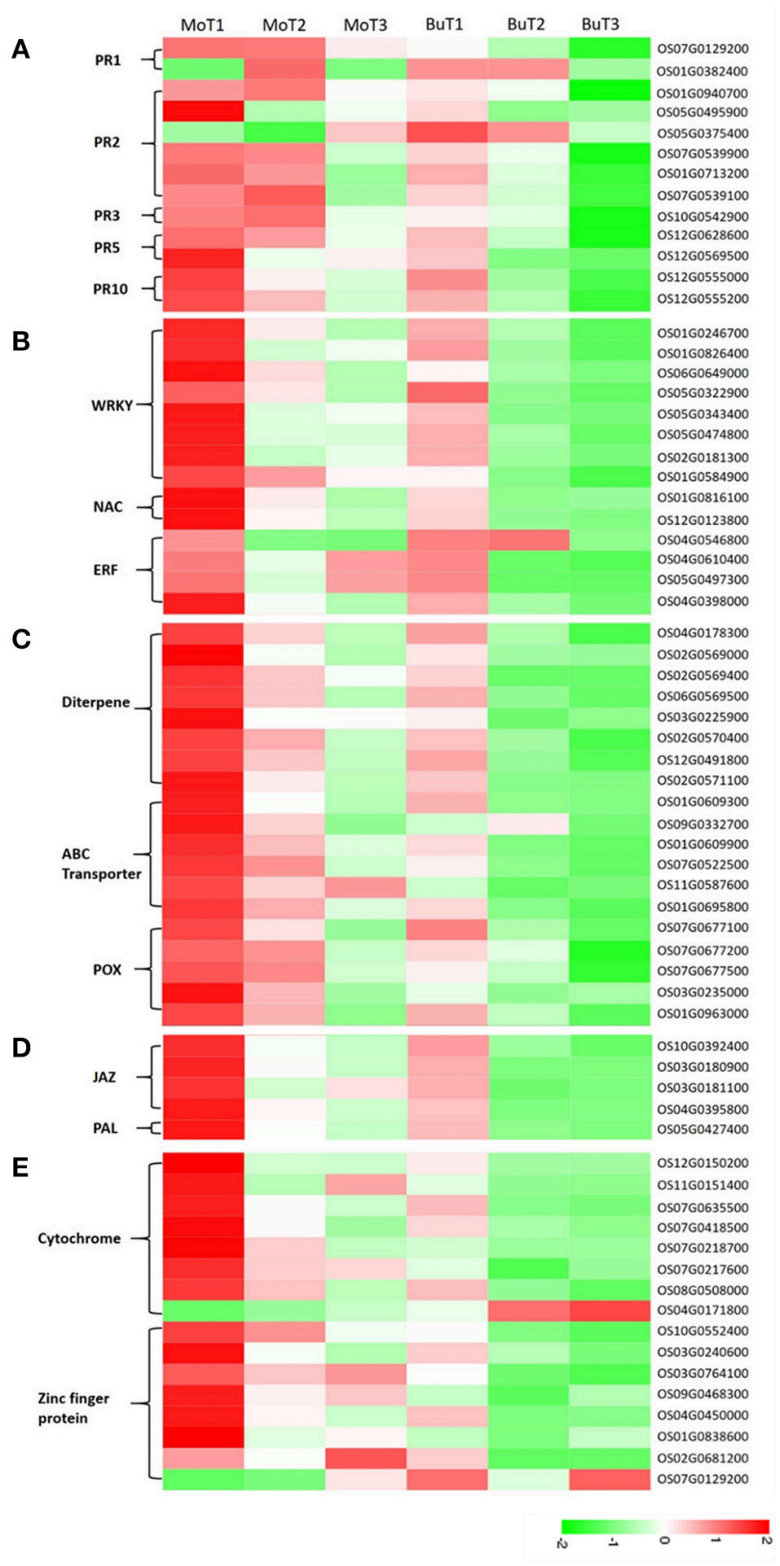
**Heat maps show increased or decreased gene transcripts in rice following MoHrip1 and buffer treatment, using the corresponding non-treated samples as controls**. The bottom color bar represents the log_10_ of the RPKM values for each gene, ranging from green (−2) to red (2). A comparison of the gene expression levels in rice after treatment with MoHrip1 and buffer for the PR proteins **(A)**; transcription factors **(B)**; phytoalexin-, ABC transporter-, and peroxidase-related genes **(C)**; SA and JA pathways **(D)**; and CYP450- and ZFP-related genes **(E)**.

The importance of pathogenesis-related proteins (PRs) in blast-resistance has been reported (Agrawal et al., 2001; Muthukrishnan et al., 2001; Hwang et al., 2007). Among the 35,679 genes detected in at least one of the 14 DGE libraries, 13 genes encode PR proteins, including 2 copies of *pathogenesis-related protein 1* (PR1), 6 β*-1-3-glucanases* (PR2), 1 *chitinase* (PR3), 2 *thaumatin-like proteins* (PR5), and 2 *prebenazole-induced genes* (PR10). Among the 2 PR1 genes, increased transcripts accumulated in the treated samples in one gene (OS07G0129200), while increased transcripts accumulated in the control samples in the other. One (OS05G0375400) of the 6 PR2 genes was induced in the control samples, and 5 genes (OS01G0940700, OS01G0940700, OS07G0539900, OS07G0539900, OS07G0539100) were induced in the treated samples. The only PR3 gene (OS10G0542900) detected was up-regulated in the treated samples and down-regulated in the control samples. As for PR5 and PR10, all genes detected (OS12G0628600, OS12G0569500, OS12G0555000, OS12G0555200) were induced both in the treated and control samples, while the level of transcript accumulation in the control samples was much lower (Figure 3A).

### WRKY, NAC, and the ERF transcription factor

Transcriptional control of the expression of resistance genes plays a crucial role in the response to stress in plants. Transcription factors have been identified as important for regulating defensive gene expression (Singh et al., 2002). In our research, WRKY, NAC, AP2/ERF were three types of transcription factors (TFs) detected, all of which were found to participate in the regulation of plant defense genes (Gutterson and Reuber, 2004; Wei et al., 2013; Sun et al., 2015). Two genes (OS06G0649000, OS01G0584900) encoding WRKY TFs were detected with increased transcript accumulation in the treated samples, while no changes were observed in the control samples. The remaining 6 genes (OS01G0246700, OS01G0246700, OS05G0322900, OS05G0343400, OS05G0474800, OS02G0181300) encoding WRKY TFs were detected in both groups but were up-regulated only in the treated groups.

The two NAC-related genes (OS01G0816100, OS01G0816100) detected were induced in the treated rice. All four genes encoding the ERF TFs were up-regulated after treatment, except for one (OS04G0546800), which was mainly down-regulated after treatment (Figure 3B).

### Antifungal-related proteins

Phytoalexins, ATP-binding cassette (ABC) transporters, and peroxidases are three types of compounds whose encoding genes were detected in our data, and all are known to be involved in reducing pathogen-related damage. Then, we classified these compounds as antifungal-related proteins. Phytoalexins play important roles in the disease resistance of various plant species, and diterpenes comprise a major portion of the phytoalexins, which are induced in rice after infection with pathogenic fungi or a pathogen-derived elicitor (Duan et al., 2014; Umemura et al., 2014). All the phytoalexin synthesis-related genes were detected in at least one treated and control group and accumulated to higher transcript levels in rice under the treatment but not the control conditions, except for one gene (OS03G0225900), which was induced in the treated group and repressed in the control group (Figure 3C).

Previous studies have shown that ABC transporters are associated with various host-pathogen interactions. The pleiotropic drug resistance (PDR) subfamily of plant ABC transporters has been implicated in plant defense (Jasiński et al., 2001; Campbell et al., 2003). In the 6 genes encoding ABC transporters (Figure 3C), half were induced and half were repressed in the rice under control conditions. However, all of the genes were up-regulated and obtained a higher level of expression in the rice after treatment than in the control rice samples.

Increased peroxidase (POX) activity has been observed in a number of resistant interactions involving plant-pathogen interactions. A previous study demonstrated that POXs were up-regulated in rice when infected by *M. oryzae* (Chittoor et al., 1997; Rauyaree et al., 2001). Five genes related to POX were detected in our data (Figure 3C). Two genes (OS07G0677500, OS03G0235000) were repressed in the control but induced in the treatment samples. The expression levels of the other 3 genes (OS07G0677100, OS07G0677200, OS01G0963000) in the groups showed that both the treatment and control conditions could induce gene expression, but that MoHrip1 produced a more intense effect in up-regulating the genes. These findings suggest that phytoalexin, the ABC transporter and POX might be involved in the resistance induced by MoHrip1 in rice.

### Salicylic acid and jasmonic acid pathways

SA and jasmonic acid (JA) are two major phytohormones responsible for regulating plant defenses against various pathogens (Bari and Jones, 2009; Thaler et al., 2012). SA biosynthesis derives from chorismate, an intermediate of plant phenylpropanoid pathway, then the phenylalanine ammonia lyase (PAL)-mediated pathway. PAL is a key regulator of the phenylpropanoid pathway and also plays an important role in regulating SA biosynthesis. The core events of JA pathway are defined after hormone perception by SCF^COI1^, JAZ (JAsmonate ZIM domain) repressors are targeted for proteasome degradation, releasing MYC2 and de-repressing transcriptional activation (Chaman et al., 2003; Chico et al., 2008). All genes encoding JAZs (OS10G0392400, OS03G0180900, OS03G0181100, OS04G0395800) had relatively low expression levels in rice under control conditions and relatively high expression levels in rice after MoHrip1 treatment. The PAL-encoding gene (OS05G0427400) has the same expression patterns in rice under the treatment and control conditions with the JAZs (Figure 3D). Therefore, it can be implied that SA pathway was activated and the JA pathway was blocked in rice treated with MoHrip1.

### Cytochrome and zinc-finger protein

The cytochrome P450 (CYP450) family is a superfamily of enzymes that participate in metabolism and form antibiotic compounds (Zhou et al., 1999; Nomura et al., 2002). In our data, eight genes related to CYP450 were detected. Four genes accumulated higher levels of transcripts in the treated rice, and these genes were induced in both the treated and control groups. Three genes were induced in the rice under treatment and were repressed in the control rice. One gene (OS04G0171800) was induced under control conditions and repressed under treatment conditions (Figure 3E).

Zinc-finger proteins (ZFPs) form a transcription factor family known to be involved in various forms of abiotic stresses, such as drought, high salinity, hot, and cold, according to many studies. However, the available research on their responses to biotic stresses remains limited (Huang et al., 2007; Islam et al., 2009). Four genes encoding ZFPs were repressed in rice under control conditions and were induced in rice under treatment conditions. The other 4 genes encoding ZFPs were induced in rice under control conditions but accumulated more transcripts in rice under treatment conditions, except for one gene (OS07G0129200), which had lower transcript levels in the treated rice (Figure 3E). The responses of CYP450 and ZFP to the elicitors indicated that these genes might be involved in contributing to the induced resistance and could be considered candidate resistance genes.

### The identification of genes induced specifically by MoHrip1 in rice

**Figure 4:**
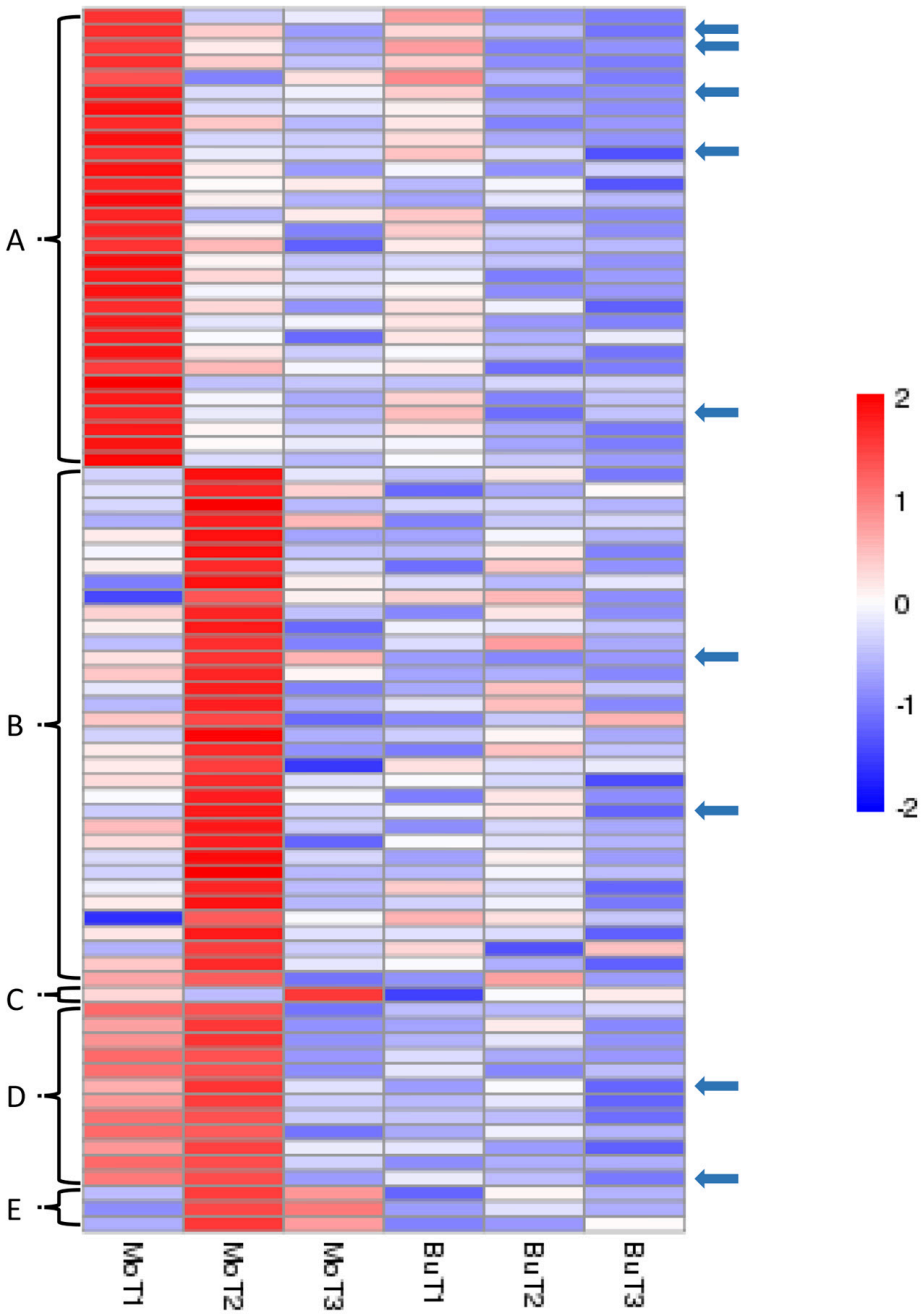
**The 80 genes with particular expression patterns in rice after treatment and low or undetected expression in rice after control treatment**. Each line indicates data for one gene. Each row indicates a cluster of genes for each library. The color bar on the right represents the log_10_ of the RPKM values for each gene, ranging from blue (−2) to red (2). **(A)**: 30 genes could be detected in MoT1 and could not be detected in any control libraries; **(B)**: 34 genes could be detected in MoT2 and could not be detected in any control libraries; **(C)**: 1 gene could be detected in MoT3 but could not be detected in the other five libraries; **(D)**: 12 genes could be detected in MoT1 and MoT2 but could not be detected in MoT3 and the control libraries; **(E)**: 3 genes could be detected in MoT2 and MoT3 but could not be detected in MoT1 and the control libraries. The arrows indicate 9 genes that were selected randomly and exhibited significantly higher gene expression levels in the treatment group than in the control groups.

In our data, we have identified 80 genes with particular expression patterns in the MoHrip1-treated group and no similar expression patterns in the buffer-treated group. These genes can be classified into five groups (Figure 4): Figure 4A, significantly induced in the MoHrip1-treated rice at 24 hat with low or undetected expression in the other conditions; Figure 4B, significantly induced in the MoHrip1-treated rice at 48 hat with low or undetected expression in the other conditions; Figure 4C, significantly induced in the MoHrip1-treated rice at 72 hat with low or undetected expression in the other conditions; Figure 4D, significantly induced in the MoHrip1-treated rice at 24 and 48 hat with low or undetected expression in the other conditions; Figure 4E, significantly induced in the MoHrip1-treated rice at 48 and 72 hat with low or undetected expression in the other conditions. These results may provide data for discovering genes that can interact with MoHrip1 in rice.

**Figure 5:**
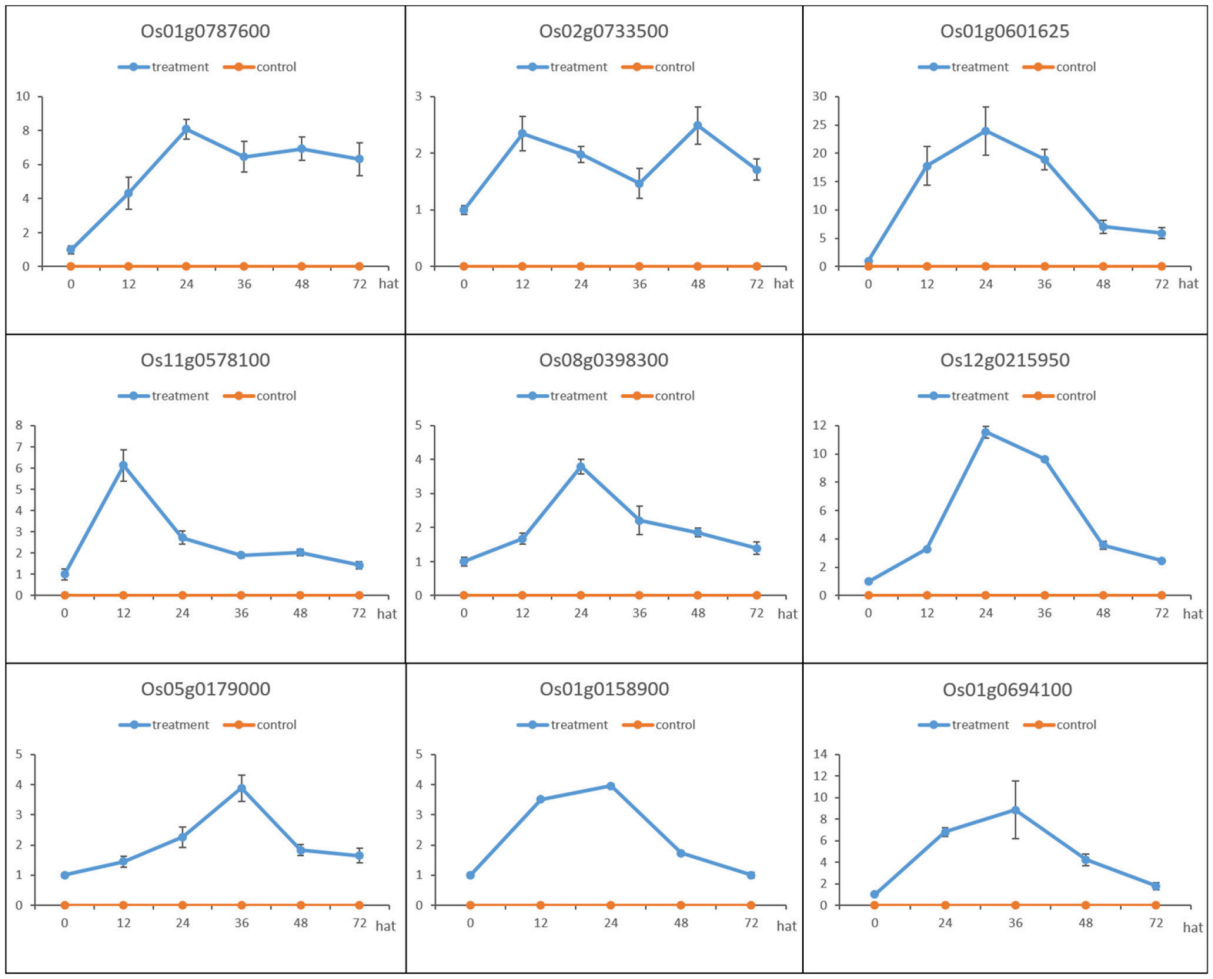
**Validation of the DGE results by qRT-PCR of the 9 genes that were up-regulated in the MoHrip1-treated rice but were not expressed in the buffer-treated rice**. The expression levels on the y-axis were relative to the non-treated rice (NoT0) after normalization with the β-actin gene. This experiment was repeated three times, and data are presented as the average ± *SD* with *n* = 3; hat: h after treatment.

Nine genes (indicated by arrows in Figure 4) that were up-regulated in MoHrip1-treated rice and not expressed in the buffer-treated rice were selected for qRT-PCR validation (Figure 5). These genes include three leucine-rich repeat proteins, an EF-hand type protein, a salicylic acid-binding protein, an ABC-transporter-like protein, two ZFPs and one heat shock protein (Table S5). The qRT-PCR results were consistent with the DGE data, as all gene expression levels in the rice under the control conditions were extremely low compared to those in the rice under treatment at certain time intervals. The 9 genes were verified as induced by MoHrip1 in rice and may be related to the resistance of rice induced by the elicitor. These genes can be selected as MoHrip1 interaction genes for further study.

### Endogenous levels of SA in rice

**Figure 6:**
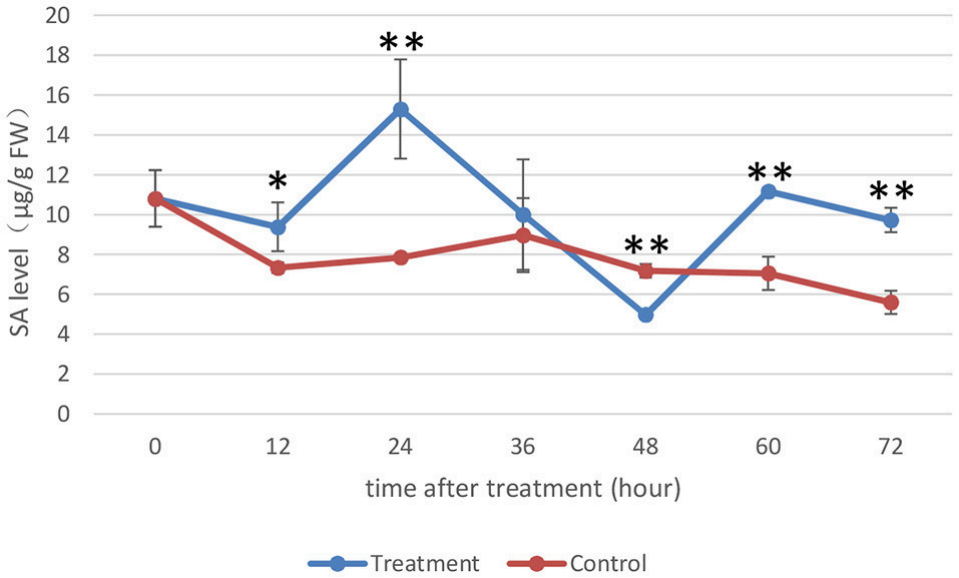
**Endogenous SA levels of rice**. The SA levels (μg/g FW) on the y-axis were relative to those of non-treated rice (NoT0). The experiment was repeated three times, and the data are presented as the average ± *SD* with *n* = 3. Asterisks indicate statistically significant differences, as measured using SAS software (Duncan's *t* test, ^**^p < 0.01, ^*^p < 0.05).

SA plays an important role in the plant immune response, and significant progress has been made in recent years in understanding the SA-mediated defense response (An and Mou, 2011). Previous research has led to a popular belief that SA and JA have antagonistic interactions (De Vleesschauwer et al., 2013). In this experiment, the SA levels in rice under the treatment and control conditions were measured as the MoHrip1-treated rice showed enhanced resistance to blast according to the results of a previous study (Figure 6). Moreover, the sequencing data indicate that the JAZ genes were induced significantly in rice under treatment conditions. JAZs were the repressors of the JA signaling and were involved in a negative regulatory feedback loop of the JA transduction pathway (Chico et al., 2008).

The SA levels detected in rice under the control conditions were not very different, while the SA levels in rice under the treatment conditions were altered significantly during the time intervals we assessed. The SA levels in MoHrip1-treated rice decreased slightly at 12 hat and increased prominently to their highest level at 24 hat. The SA levels started to decrease at 36 and 48 hat, began to increase at 60 hat and finally decreased again at 72 hat. Intriguingly, the SA levels of the rice under the treatment conditions across all time intervals were higher than those of the rice under control conditions, excluding 48 hat, at which the SA levels reached their lowest point. This result can be explained through the feedback regulation of SA, which was likely induced significantly by MoHrip1.

### Endogenous levels of GA in rice and growth promotion

**Figure 7:**
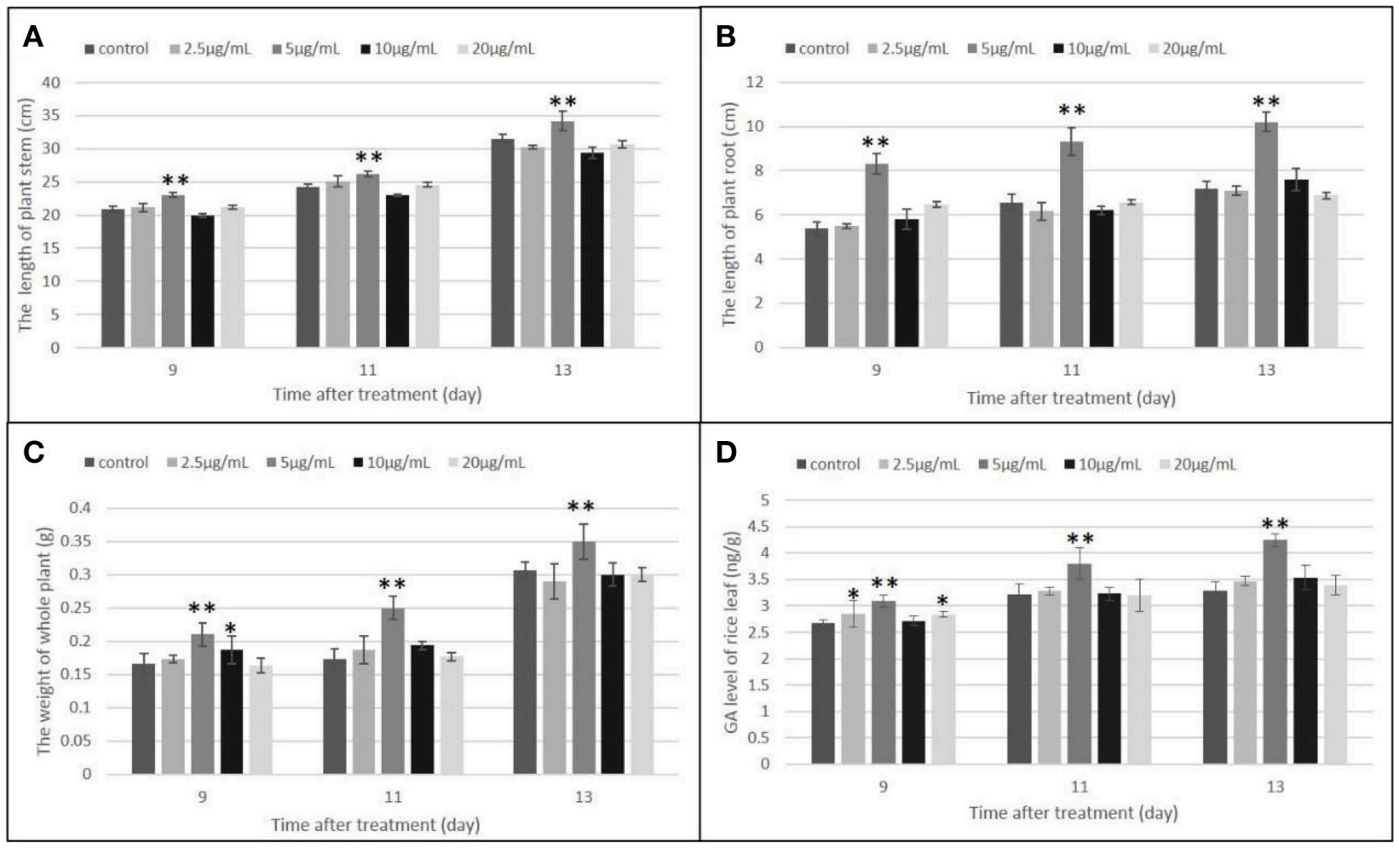
**Growth promotion and GA level of plant seeds treated with a range of concentrations of MoHrip1**. **(A)** Average length of the plant stem. **(B)** Average length of the plant root. **(C)** Average weight of the whole plant. **(D)** GA levels of plants under different treatment conditions. Values are presented as the mean ± *SD* of three replicates, each consisting of 15 seedlings. Asterisks indicate statistically significant differences, as measured using SAS software (Duncan's *t* test, ^**^p < 0.01, ^*^p < 0.05).

**Figure 8:**
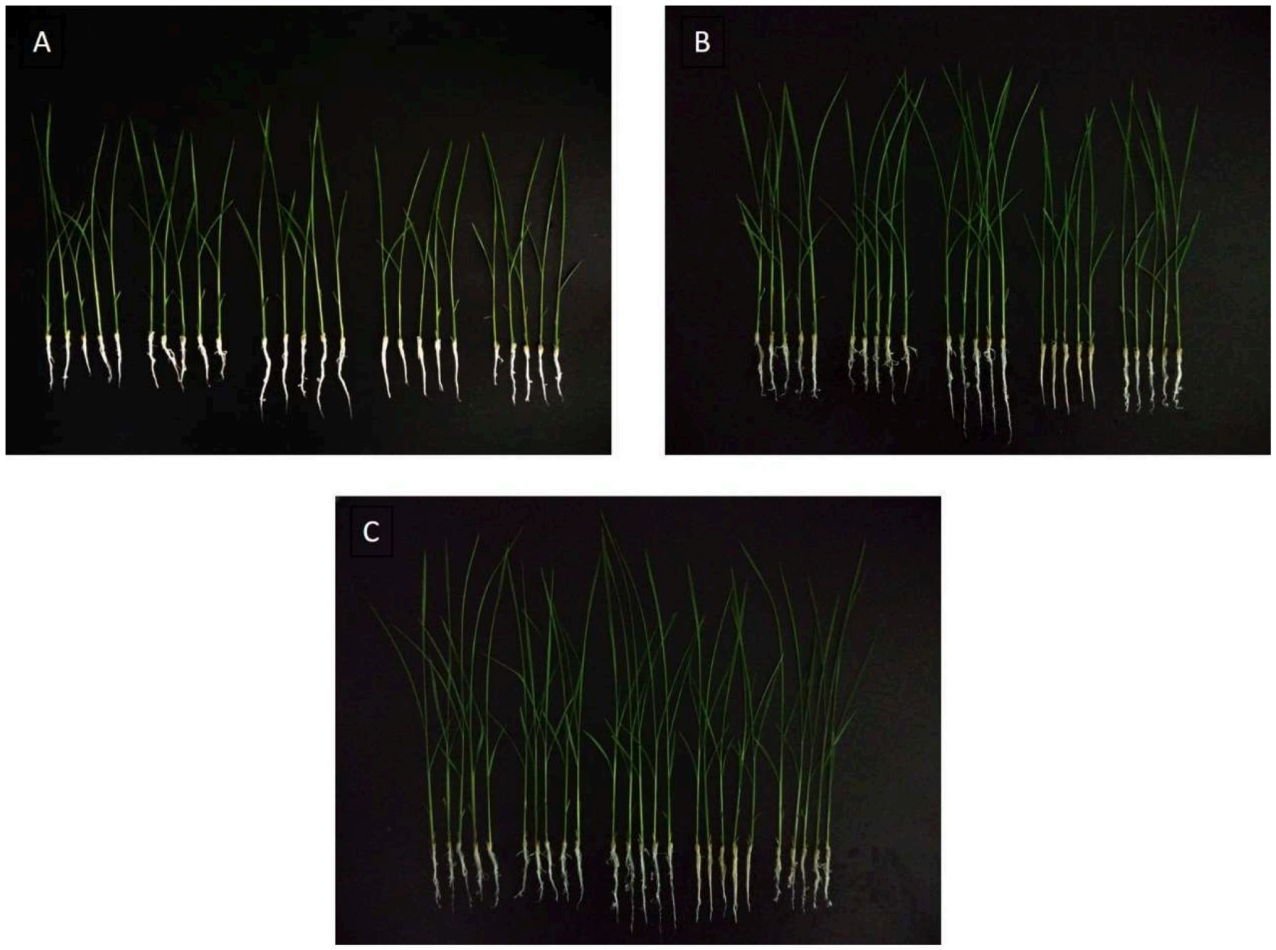
**Rice seeds treated with a range of concentrations of MoHrip1 show significant differences in their growth morphological characterization**. **(A)** Rice seed germination after 9 days of treatment. **(B)** Rice seeds germinated after 11 days of treatment. **(C)** Rice seeds germinated after 13 days of treatment. The group arranged horizontally in each graph indicates the different concentrations of MoHrip1 used to treat the rice seeds (0, 2.5, 5, 10, and 20 μg/mL, from left to right).

Gibberellins (GAs) are a type of essential phytohormone and control many aspects of plant growth and development. Recent studies have revealed that crosstalk between GA and JA signaling is involved in both plant development and defense. Moreover, the crosstalk is regulated by interaction between the DELLA and JAZ proteins as repressors to modulate the activity of relative transcriptional factors in response to the GA and JA signals (Hou et al., 2013). We predicted that the induced JAZs might indirectly affect GA levels. The endogenous GA levels were then measured, along with the length and weight of the rice under the treatment and control conditions (Figures 7, 8). All measured data indicated that 5 μg/ml was the best concentration of MoHrip1 to produce the greatest effect on promoting the growth of rice. According to this experiment, the morphological differences observed at each concentration of MoHrip1 started 9 days after treatment (Figures 7A–C, 8). Moreover, the phytohormone GA also required 9 days for growth promotion to be observed at a significant level (Figure 7D). These results indicate that MoHrip1 may influence the level of GA and that there is an optimum concentration at which GA is most effective in regulating plant growth.

### Induction of resistance to rice blast by MoHrip1

**Figure 9:**
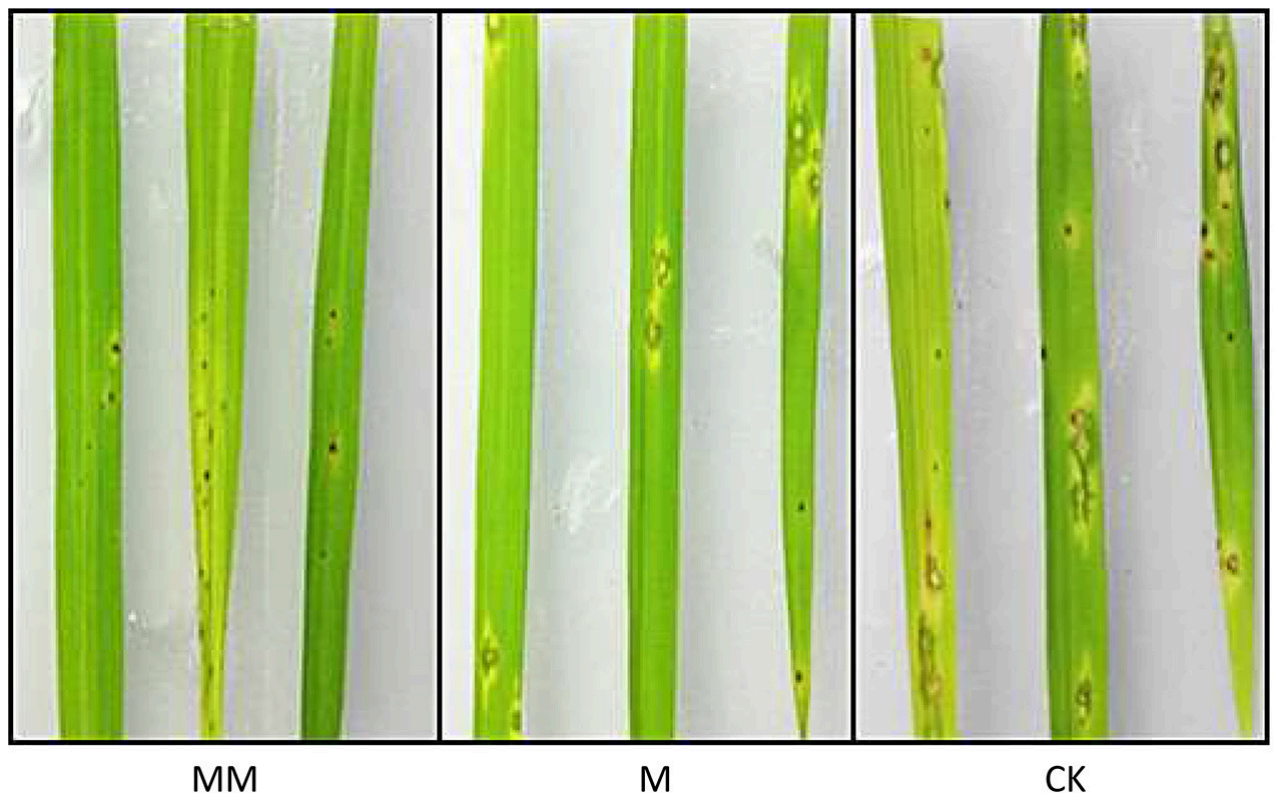
**Typical disease symptoms on leaves of MoHrip1-treated and control-treated rice plants**. Rice plants were sprayed with *M. oryzae* spores after being treated with MoHrip1 or Tris-Cl 3 days. The MM group indicated that the rice seeds were immersed in MoHrip1 (10 μg/mL) then sprayed with MoHrip1 (30 μM) at the three-leaf stage. The M group indicated that the rice seeds were immersed in MoHrip1 (10 μg/mL) then sprayed with Tris-Cl (25 mM) at the three-leaf stage. The CK group indicated that the rice seeds were immersed in H_2_O then sprayed with Tris-Cl (25 mM) at the three-leaf stage as a negative control.

Three groups of rice plants were sprayed with *M. oryzae* spore suspension (strain KJ201) after 3 days of incubation with either MoHrip1 or Tris-Cl. Screening was carried out on detached leaves. The lesions on the leaves of CK group exhibited typical and severe symptoms at 7 days after inoculation. At this time, only small and constrained lesions were observed on the leaves of both MM group and M group, and most of the plants remained green and healthy. While the constrained lesions on leaves of MM group were smaller than those on leaves of M group (Figure 9). The disease severity of all plants from the three groups was evaluated on a standard international 0–9 scale at 7 days post-inoculation, the leaf blast score of MM was a little lower than that of M group. While the blast score of MM group and M group was much lower than that of CK group (Table 3). Furthermore, we measured the root length of rice plants after we examined the disease indices. MM group and M group exhibited obvious function of MoHrip1 in growth promotion (Figure S8). Taken together, the results from the disease-resistance assay and growth promotion assay reconfirmed the inference that MoHrip1 could not only increase rice plant resistance to the rice blast but also promote plant growth.

**Table 3:** **Disease severity of rice blast in leaves of MoHrip1-treated and control-treated rice plants**.

**Rice seedling samples**	**Rice blast score**
MM (Rice immersed in MoHrip1 and sprayed with MoHrip1)	3.126 ± 0.212Aa
M (Rice immersed in MoHrip1 and sprayed with Tris-Cl)	3.734 ± 0.173Ab
CK (Rice immersed in H_2_O and sprayed with Tris-Cl)	5.862 ± 0.285B

*The disease scores of the rice seedlings were evaluated on a scale of 0–9 at 7 days post-inoculation. Values are presented as the mean ± SD of three replicates, each consisting of 25 seedlings. Values with different letters indicate statistically significant differences (Duncan's t test, different capital letters means p < 0.01, different small letters means p < 0.05)*.

## Discussion

### Advantages and defects of DGE in the gene expression profiling study

In the early stage of studies, elicitor-responsive genes were identified by means of differential mRNA display analysis, DNA microarrays, and other methods (Kim et al., 2000; Day et al., 2002). These methods were later replaced by NGS-based transcriptome profiling, which is based on the sequencing of relatively short reads, with extensive sequence data to allow for the detection and quantification of rare or novel gene discovery (Morrissy et al., 2009). DGE, as used in this study, includes profiles based on NGS, generates digital rather than simulated gene expression measurements and avoids many of the inherent limitations of other analysis methods (Xiao et al., 2013).

DGE has already been used for studying genes in many species, such as wheat, sweet potato, and rice (Tao et al., 2012; Xiao et al., 2013). In general, current studies aiming at resistance-related genes in rice, which introduced NGS-based technology, use two styles of research model: a pathogen vs. whole plant model or a pathogen secretion vs. rice suspension cell model (Day et al., 2002; Bagnaresi et al., 2012). In this study, MoHrip1, a protein elicitor secreted by *M. oryzae*, and the whole rice plant form a new style of research model. We obtained sufficient data from the Illumina sequencing experiment (Table 1) to allow for gene detection and applied real-time PCR to validate the DGE veracity (Figure 1). A total of 308 genes were differentially expressed, and 80 genes were expressed in the treatment condition but not in the control condition. These findings indicate that pathogen secretion (elicitor) vs. the whole plant could constitute a third research model and that DGE could fulfill the technical requirements needed to identify potentially interesting genes. This technology also has its drawbacks. DGE was a method designed to identify DEGs based on relatively short sequence reads. Therefore, the differences between the experimental and control groups are necessary; genes with similar expression levels in the groups but acted functionally might be ignored. In conclusion, our results provide an overview of the gene expression patterns of elicitor-treated rice and offer a valuable set of data for the further discovery of candidate genes that interact with the elicitor.

### SA played a major role in the blast-resistance of rice and may be regulated by MoHrip1

*M. oryzae* is a hemibiotrophic pathogen, as its invasion begins with a biotrophic phase followed by necrotrophic phase, leading to host cell death (Talbot, 2003). In general, the JA pathway is associated with defense against necrotrophic pathogens, while the SA pathway is thought to be involved in the plant resistance to biotrophic and hemibiotrophic pathogens (Bari and Jones, 2009). The positive role of the SA pathway in blast-resistance has been reported (Iwai et al., 2007; Daw et al., 2008). The SA pathway includes two steps: SA biosynthesis followed by SA signal transduction, which leads to the activation and expression of SA-responsive genes (Ellis and Amrhein, 1971; Zhu-Salzman et al., 1998). The JA pathway possesses two similar steps to the SA pathway (Fonseca et al., 2009).

**Figure 10:**
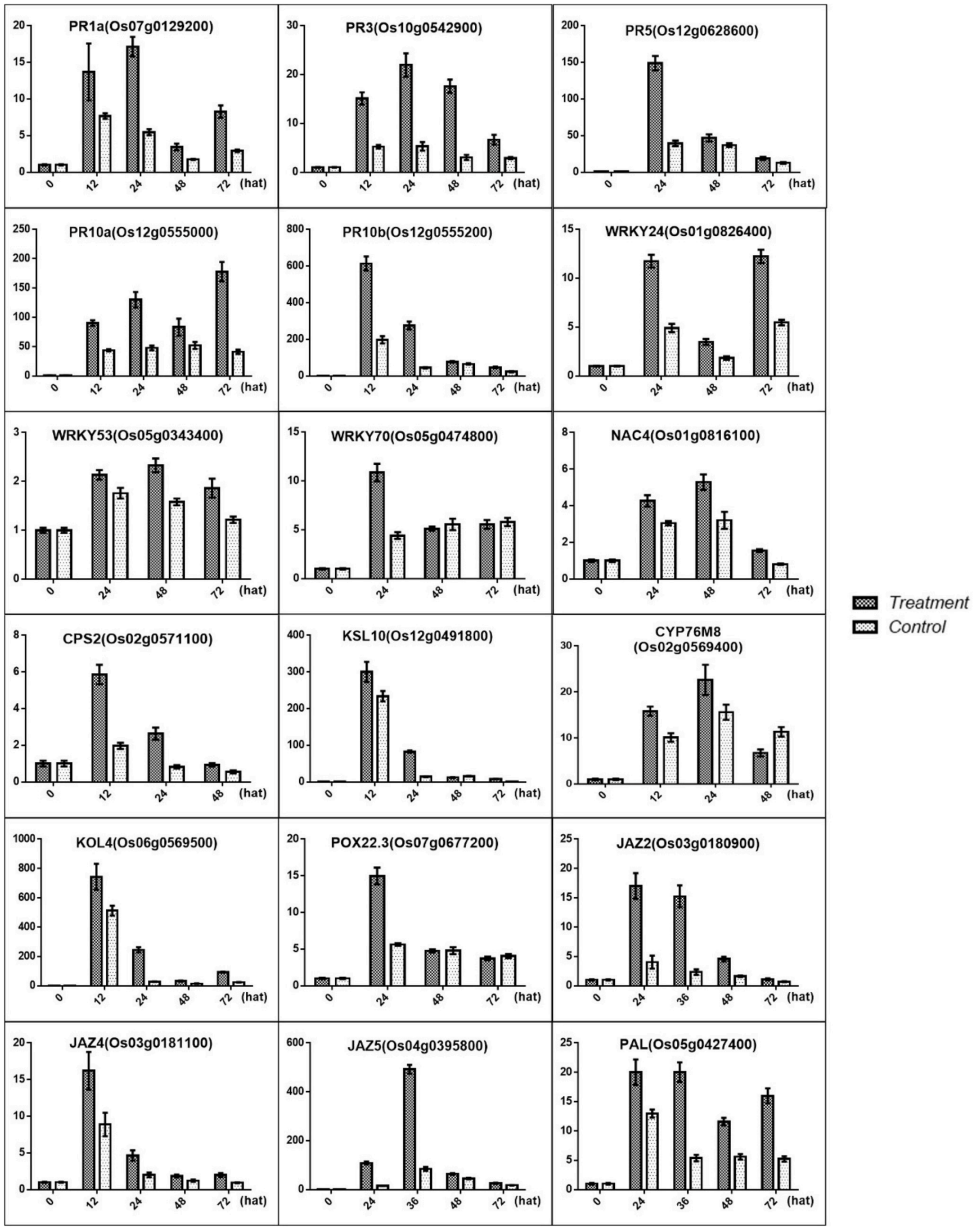
**Validation of the DGE transcriptome results by qRT-PCR for the genes involved in the resistance induced by MoHrip1 in rice**. The expression levels on the y-axis were relative to those of the non-treated samples (0 h) after normalization with the rice β-actin gene. The experiment was repeated three times, and the resulting data are presented as the average ± *SD*, with *n* = 3; hat: h after treatment.

Based on our data, the SA biosynthesis pathway was activated, and the result was confirmed by qRT-PCR for PAL (OS05G0427400), which was involved in SA biosynthesis. In contrast, the JA signal transduction pathway was blocked, and this result was confirmed by qRT-PCR for the JAZ2 (OS03G0180900), JAZ4 (OS03G0181100), and JAZ5 (OS04G0395800) repressors of JA signal transduction (Figure 10). One SA biosynthesis-associated gene and 3 JA pathway repressor genes were significant differentially expressed. As the antagonistic function hypothesis for the SA-JA crosstalk research prevailed, and the endogenous SA levels in the treated condition were obviously higher than those in the control condition, it can be predicted that SA biosynthesis was induced in rice treated with MoHrip1.

Previous studies have identified some PR genes involved in pathogen resistance located downstream of the SA pathway that are regulated by the SA signals (Agrawal et al., 2001; McGee et al., 2001; Rakwal et al., 2001). These PR genes were also found to be up-regulated, and this result was confirmed by qRT-PCR for PR1a (OS07G0129200), PR3 (OS10G0542900), PR5 (OS12G0628600), PR10a (OS12G0555000), and PR10b/PBZ1 (OS12G0555200) (Figure 10). Furthermore, transcription factors were another important part of regulators involved in pathogen resistance. Some TFs were found to act as a node between the SA and JA pathway, which activates SA-induced genes and represses JA-induced genes (Li et al., 2004; Shimono et al., 2007). Some TFs are involved in the plant defense response along with SA (Kaneda et al., 2009). Some TFs associated with SA were induced in rice under treatment conditions, and the results were verified by qRT-PCR for WRKY24 (OS01G0826400), WRKY53 (OS05G0343400), WRKY70 (OS05G0474800), and NAC4 (OS01G0816100) (Figure 10).

An increasing number of studies has shown that rice possesses a high basal level of SA. Although the primary function of SA may not be to induce the expression of defense genes, it still appears to protect rice from damage during pathogen infection (Yu et al., 1997; Yan and Dong, 2014). From our results, even though the SA content increased, no significant difference was noted in the rice in the treatment group compared with the rice in the control group. Furthermore, a large amount of resistance-related substances, which were associated with SA, were obviously accumulated. It can be assumed that an increased level of SA may not only provide an effective signal to induce gene expression but also play an important role in reducing pathogen-induced damage. Moreover, our results verified the importance of the SA pathway in blast-resistance.

### Phytoalexin may be important in blast-resistance induced by MoHrip1

Phytoalexins include a range of secondary metabolites produced in rice upon *M. oryzae* infection that function in the defense system (Wang et al., 2012; Miyamoto et al., 2014). The rice phytoalexins have been identified to fall into two groups, and the diterpenoid-type phytoalexins formed the major portion of these substances. All phytoalexin-related genes that were up-regulated in our data were involved in the biosynthesis of phytocassanes A–E and oryzalexin A–F. Oryzalexin is considered a biologically important phytoalexin, as it has high antimicrobial activity in *in vitro* assays. There is no direct evidence to prove its function in blast-resistance. The two types of phytoalexin shared the same precursor. Our results showed that phytoalexin biosynthesis related genes were increased in rice after treatment with MoHrip1, and the results were confirmed by qRT-PCR for CPS2 (OS02G0571100), KSL10 (OS12G0491800), KOL4 (OS06G0569500), and CYP76M8 (OS02G0569400). These findings suggest that phytoalexins, especially Oryzalexin, may be important in the blast-resistance induced by MoHrip1 and that phytocassanes may be involved in the defense response.

### GA may be induced to promote plant growth through the crosstalk between Della and Jaz, which is induced by MoHrip1

GAs are plant hormones that play pivotal roles in promoting growth. Current research suggests that GA promotes plant growth by regulating the degradation of DELLAs, a class of nuclear growth repressors. High levels of DELLAs will provide feedback that negatively controls GA levels, while low levels of DELLAs may stimulate GA production to obtain a balance between the GAs and DELLAs (Claeys et al., 2014). Previous studies have demonstrated that the GA-signaling repressor DELLA and the JA-signaling repressor JAZ engage in crosstalk that modulates the action between GA and JA to regulate plant growth (Yang et al., 2012). According to our data, the JAZs are induced by MoHrip1, followed by more extensive crosstalk between the JAZs and DELLAs. Although the level of DELLAs was not measured, it can be predicted that increased JAZs led to DELLA repression based on the GA contents measured in this experiment, resulting in an overall growth promotion in rice. The results were confirmed by qRT-PCR (Figure 10) and morphologic testing (Figure 7).

Intriguingly, the GA and phytoalexin synthesis pathways shared the same precursors: geranylgeranyldiphosphate (GGDP) and *ent*-copalyldiphosphate (*ent*-CPP). However, there appears to be separate sets of *ent*-CPP synthases for gibberellin (KS1) vs. phytoalexin (KSL7, KSL10) biosynthesis. There was no evidence to demonstrate that KS1 was induced in the rice after treatment, while the genes encoding KSL7 and KSL10 were confirmed to be up-regulated under treatment. These findings suggested that the increased GA levels did not result from the sequential catalysis derived from GGDP and were instead affected by the crosstalk between the GA and JA pathway repressors caused by MoHrip1.

## Conclusion

We present an RNA-seq study focusing on the response of rice to secretion protein elicitors. Our transcriptome data provide comprehensive insight into the gene expression profiles induced by MoHrip1 in rice. SA was the main phytohormone induced to participate in blast-resistance, and the JA pathway was repressed in rice after treatment with MoHrip1. Phytoalexins were induced to synthesized and played an important role in reducing the damage caused by pathogen infection. The GA content increased to promote plant growth after rice was treated with MoHrip1. There was a balance and exchange between the plant defense response and plant growth promotion. The DGE data revealed that the protein elicitor MoHrip1 possessed the potential not only to protect plants by inducing pathogen-resistance but also to regulate plant growth indirectly, even though involvement with some other aspects of the plant life cycle. Nine genes were identified as candidates, and it would be of great value to further characterize their roles in interactions with MoHrip1.

## Author contributions

SL, ZW, and HZ designed research; SL and ZW performed research; SL analyzed data and wrote the paper; XY, LG, DQ, and HZ revised this paper.

## Funding

This work was supported by grants from the National Hi-Tech Research and Development Program of China (“863” Projects, Grant Nos. 2012AA101504).

### Conflict of interest statement

The authors declare that the research was conducted in the absence of any commercial or financial relationships that could be construed as a potential conflict of interest.

## Supplementary material

Figure S1 **Saturation curve of DGE sequencing**. Quantified gene expression with 10, 20, 30……90% of the total mapped reads as the final scores. Comparing the RPKM of one gene in each score with the final gene expression level means that genes can be quantified accurately in this score when the deviation is less than 15%. The X-axis represents the percentage of reads mapping to the genome, while the Y-axis represents the ratio of genes with a qualify error of 15%. The pictures from **(A–N)** represent NoT0_1, NoT0_2, MoT1_1, MoT1_2, BuT1_1, BuT1_2, MoT2_1, MoT2_2, BuT2_1, BuT2_2, MoT3_1, MoT3_2, BuT3_1, and BuT3_2, respectively.

Figure S2 **Pearson correlation coefficients between samples**. The axis represents the gene expression levels of one sample in two replicates under the same condition. **(A–G)** represent the correlations between the two replicates of the NoT0, BuT1, MoT1, BuT2, MoT2, BuT3, and MoT3 groups, respectively.

Figure S3 **Histogram of GO enrichment**. The x-axis indicates the number of genes enriched in terms, while the y-axis indicates the enriched GO terms. Different colors represent biological process (green), cell component (blue) and molecular function (red). ^*^Indicates GO terms with significant enrichment. **(A–C)** Represent the GO enrichments of the differentially expressed genes in rice after 1, 2, and 3 days of treatment, respectively.

Figure S4 **Scatter diagram of the KEGG enrichment**. The y-axis indicates the name of the pathway, and the x-axis represents the Rich factor. The spot size indicates the number of differentially expressed genes in the pathway. The *q*-value represents the corrected *p*-value, and a *q* < 0.05 indicates significant enrichment. **(A–C)** Represent the pathway enrichment of differentially expressed genes in rice after 1, 2, and 3 days of treatment, respectively.

Figure S5 **Volcano plot**. The volcano plot indicates the expressed genes, and red or green spots indicate genes with significant expression differences. Red indicates up-regulated genes, while green indicates down-regulated genes. Blue spots indicate genes without significant expression differences. The x-axis indicates the fold change in the gene expression levels in the different samples. The y-axis indicates the statistical significance of the gene expression differences. MoT1 vs. BuT1, MoT2 vs. BuT2, and MoT3 vs. BuT3 represent the comparison between the treatment and control rice groups after MoHrip1 treatment for 1, 2, and 3 days.

Figure S6 **Venn diagrams of the groups. (A)** Differentially expressed genes among the three groups of treated and control samples superimposed. **(B,C)**, numbers of differentially expressed genes in the treated and control samples compared with the non-treated samples. NoT1, MoT1, BuT1, MoT2, BuT2, MoT3, BuT3 represent the 0-day non-treatment group, the 1-day treatment group, the 1-day control group, the 2-day treatment group, the 2-day control group, the 3-day treatment group, and the 3-day control group, respectively.

Figure S7 **Volcano plots show that the treated rice samples have more differentially expressed genes relative to the non-treated rice samples than the control rice samples**. MoT1 vs. NoT0, MoT2 vs. NoT0, and MoT3 vs. NoT0, and MoT3vsNoT0 represent the comparisons between the treated rice and the non-treated rice after MoHrip1 treatment for 1, 2, and 3 days. BuT1 vs. NoT0, BuT2 vs. NoT0, and BuT3 vs. NoT0 represent the comparisons between the control and non-treated rice groups after treatment with protein buffer for 1, 2, and 3 days.

Figure S8 **Root growth promotion of rice after treated with MoHrip1 or Tris-Cl. (A)** Root growth promotion of rice plants. **(B)** Average length of rice plant root. MM (Rice immersed in MoHrip1 and sprayed with MoHrip1), M (Rice immersed in MoHrip1 and sprayed with Tris-Cl), and CK (Rice immersed in H_2_O and sprayed with Tris-Cl) were measured after 7 days inoculation with *M. oryzae* spores. Values are presented as the mean ± SD of three replicates, each consisting of 25 seedlings. Asterisks indicate statistically significant differences, as measured using SAS software (Duncan's *t* test, ^**^*p* < 0.01, ^*^*p* < 0.05).

Table S1 **Primer sequences used in this study**.

Table S2 **Statistics of the pathway enrichment of differentially expressed genes detected in treated rice after 1 day**. A corrected *p* < 0.05 represents statistically significant enrichment of that pathway.

Table S3 **Ct and Log_2_ RPKM values for selected genes**. Genes were designated as “undetected” when RPKM = 0.01.

Table S4 **List of the gene IDs and their functional descriptions from two detailed gene clustering analyses**.

Table S5 **Eighty genes with particular expression patterns in the treated rice and low or undetected expression in the control rice**. Gene IDs with bold fonts were selected for qRT-PCR validation.
