# Supplementary figures and images for: Transcriptional Profiling of Rice Treated with MoHrip1 Reveal the Function of Protein Elicitor in Enhancement of Disease Resistance and Plant Growth
Source: Front Plant Sci. 2016 Dec 1;7:1818. doi: 10.3389/fpls.2016.01818 (PMC5131010; doi:10.3389/fpls.2016.01818)

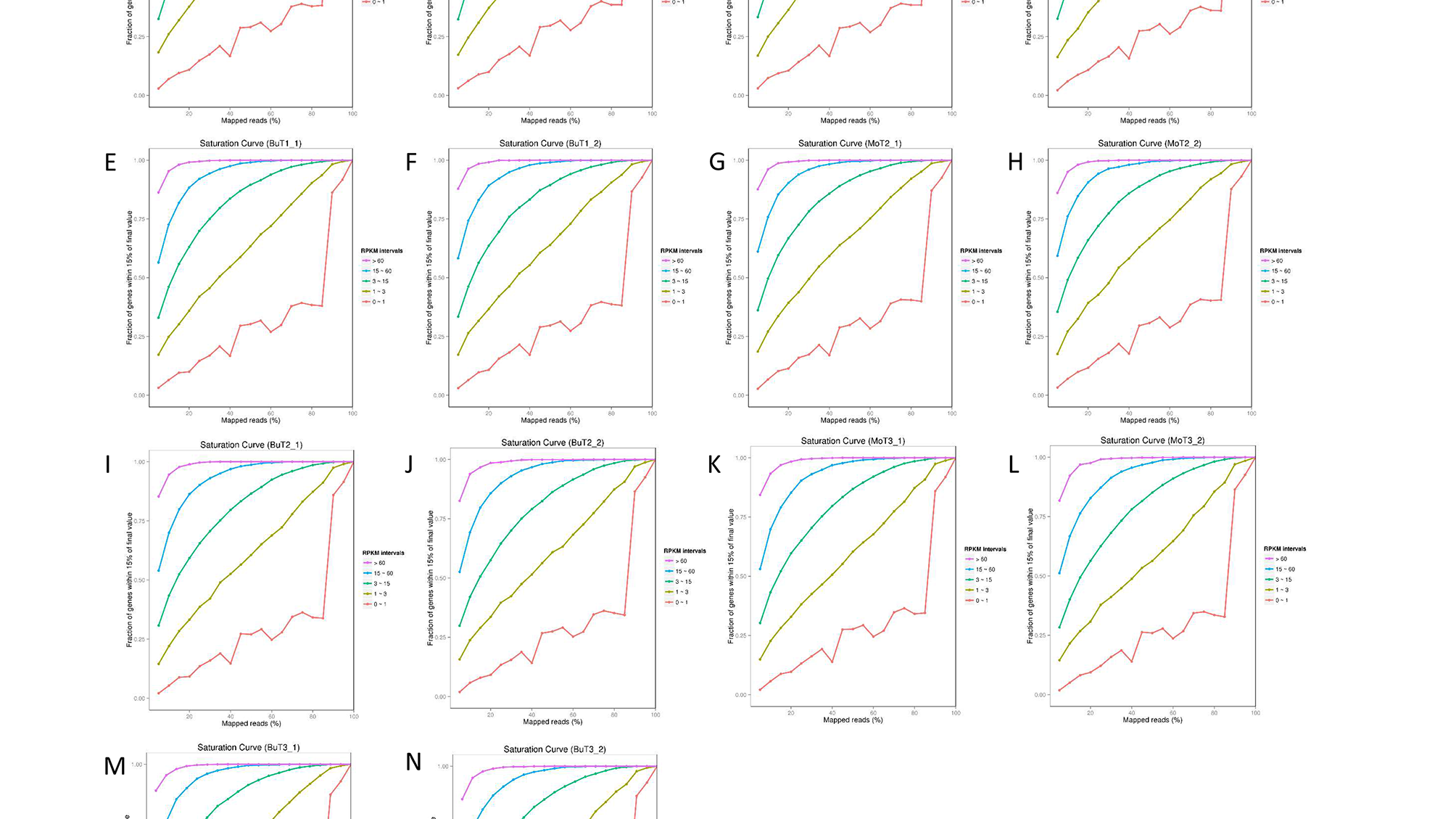

Supplement: Figure S1 — Saturation curve of DGE sequencing. Quantified gene expression with 10, 20, 30……90% of the total mapped reads as the final scores. Comparing the RPKM of one gene in each score with the final gene expression level means that genes can be quantified accurately in this score when the deviation is less than 15%. The X-axis represents the percentage of reads mapping to the genome, while the Y-axis represents the ratio of genes with a qualify error of 15%. The pictures from (A–N) represent NoT0_1, NoT0_2, MoT1_1, MoT1_2, BuT1_1, BuT1_2, MoT2_1, MoT2_2, BuT2_1, BuT2_2, MoT3_1, MoT3_2, BuT3_1, and BuT3_2, respectively. [file Image1.TIF]

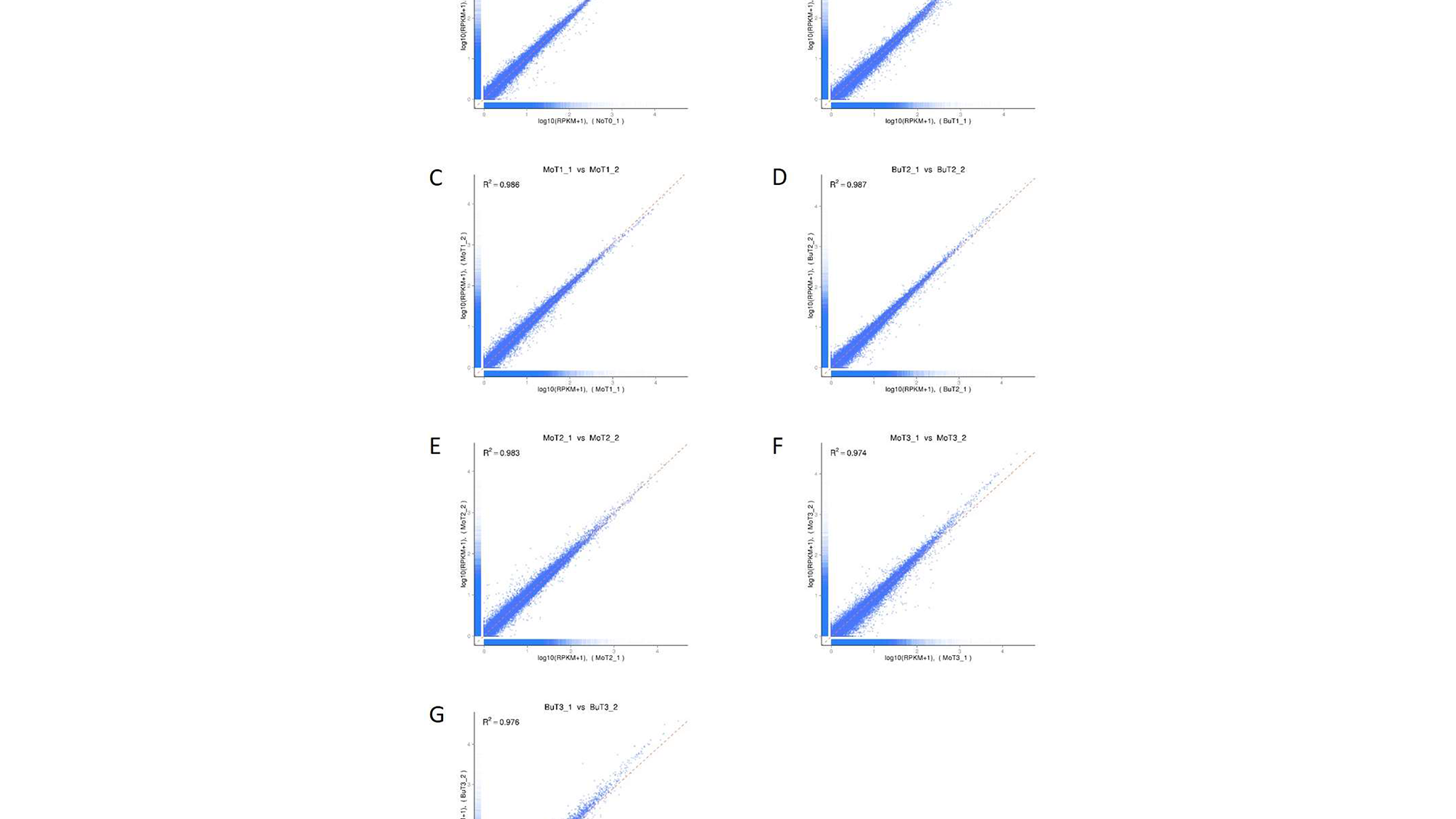

Supplement: Figure S2 — Pearson correlation coefficients between samples. The axis represents the gene expression levels of one sample in two replicates under the same condition. (A–G) represent the correlations between the two replicates of the NoT0, BuT1, MoT1, BuT2, MoT2, BuT3, and MoT3 groups, respectively. [file Image2.TIF]

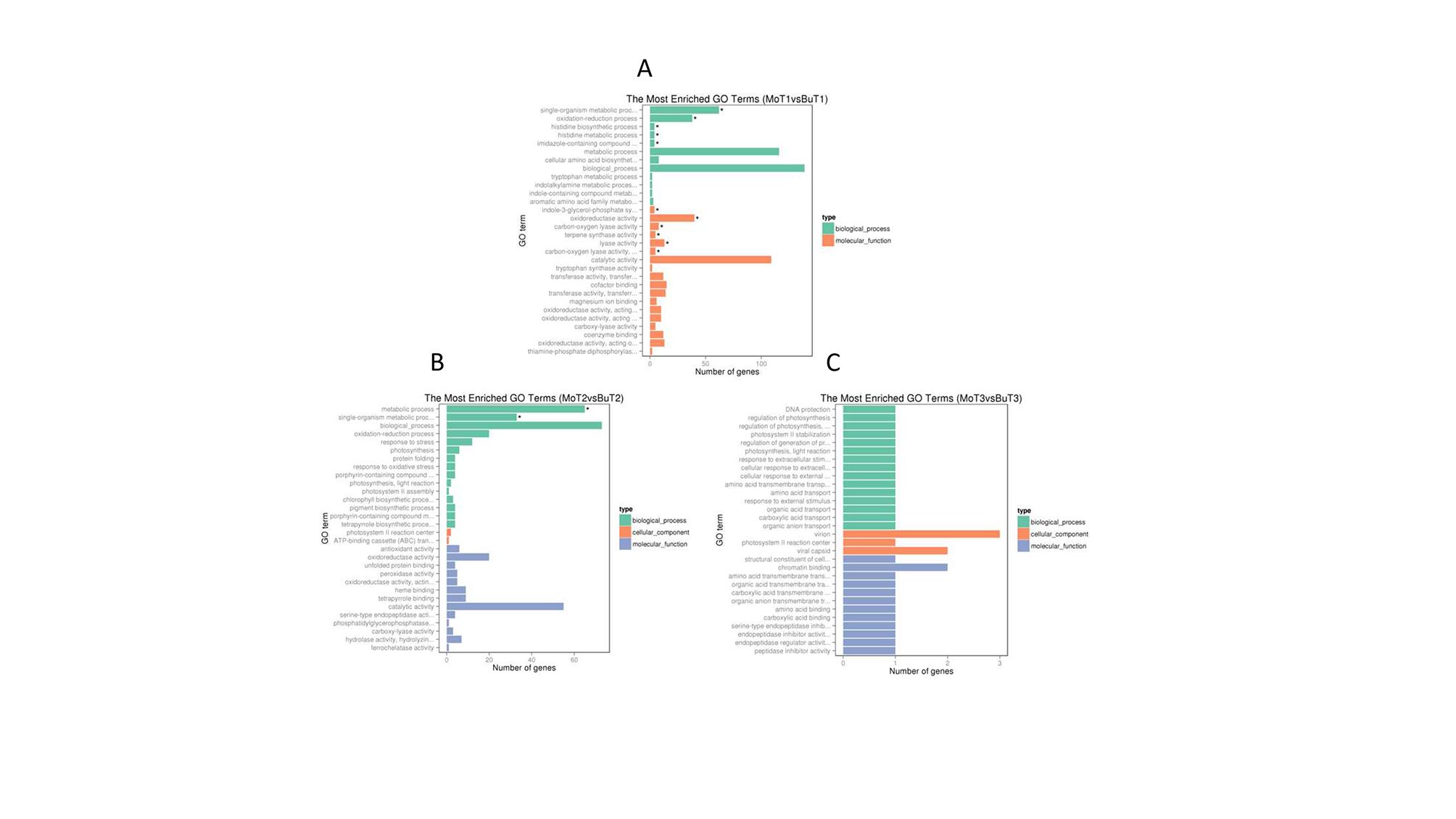

Supplement: Figure S3 — Histogram of GO enrichment. The x-axis indicates the number of genes enriched in terms, while the y-axis indicates the enriched GO terms. Different colors represent biological process (green), cell component (blue) and molecular function (red). *Indicates GO terms with significant enrichment. (A–C) Represent the GO enrichments of the differentially expressed genes in rice after 1, 2, and 3 days of treatment, respectively. [file Image3.TIF]

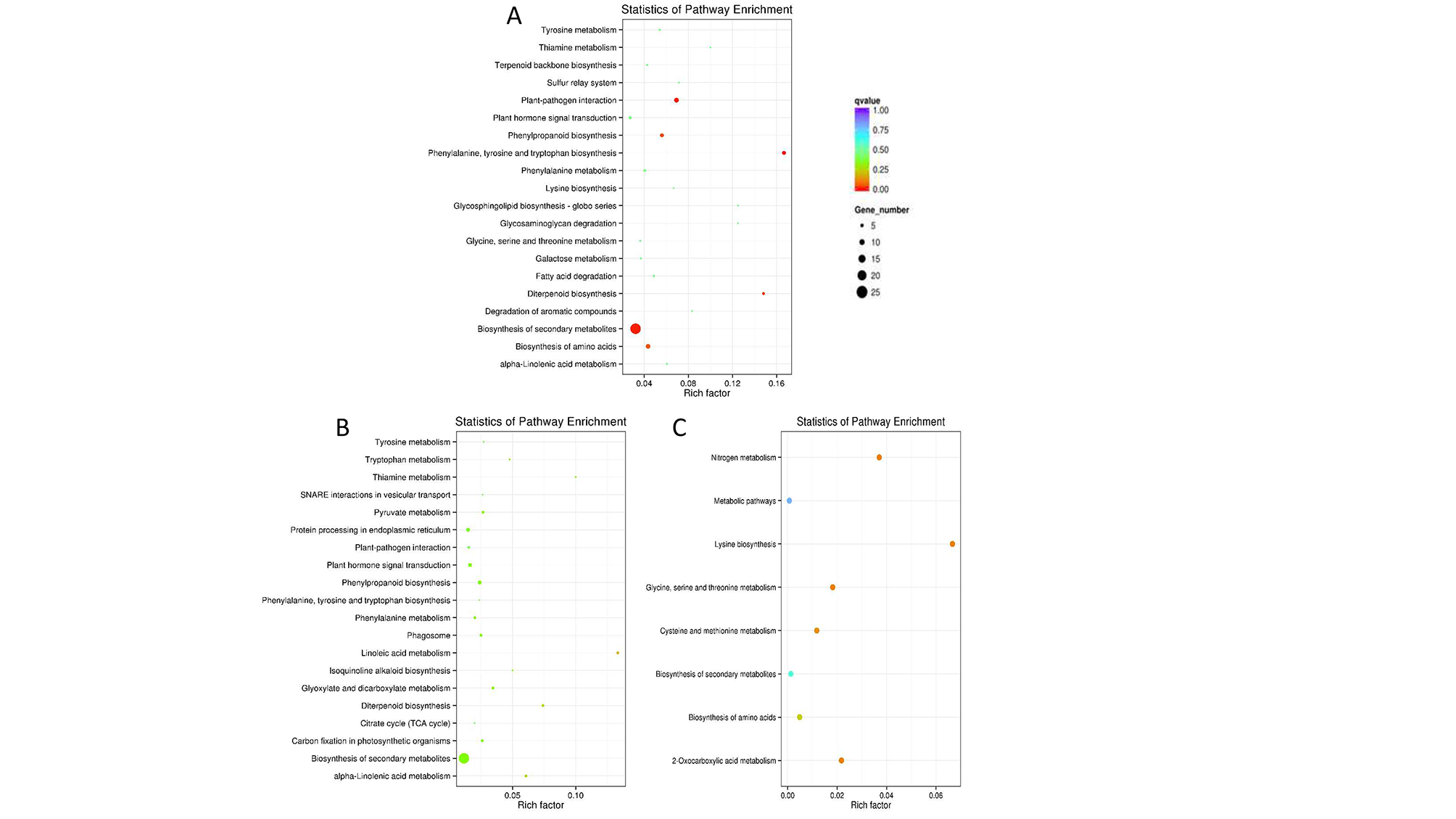

Supplement: Figure S4 — Scatter diagram of the KEGG enrichment. The y-axis indicates the name of the pathway, and the x-axis represents the Rich factor. The spot size indicates the number of differentially expressed genes in the pathway. The q-value represents the corrected p-value, and a q < 0.05 indicates significant enrichment. (A–C) Represent the pathway enrichment of differentially expressed genes in rice after 1, 2, and 3 days of treatment, respectively. [file Image4.TIF]

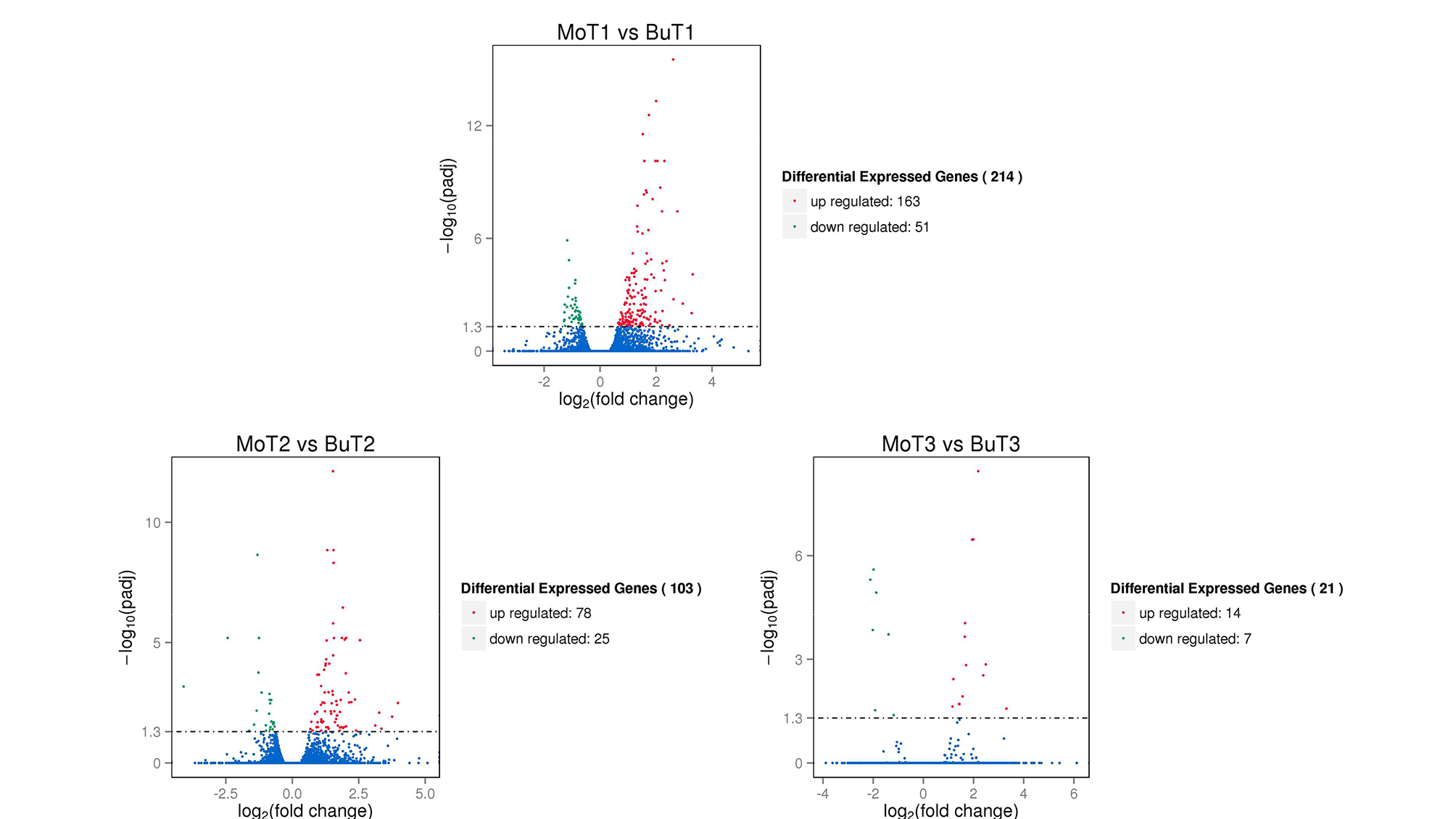

Supplement: Figure S5 — Volcano plot. The volcano plot indicates the expressed genes, and red or green spots indicate genes with significant expression differences. Red indicates up-regulated genes, while green indicates down-regulated genes. Blue spots indicate genes without significant expression differences. The x-axis indicates the fold change in the gene expression levels in the different samples. The y-axis indicates the statistical significance of the gene expression differences. MoT1 vs. BuT1, MoT2 vs. BuT2, and MoT3 vs. BuT3 represent the comparison between the treatment and control rice groups after MoHrip1 treatment for 1, 2, and 3 days. [file Image5.TIF]

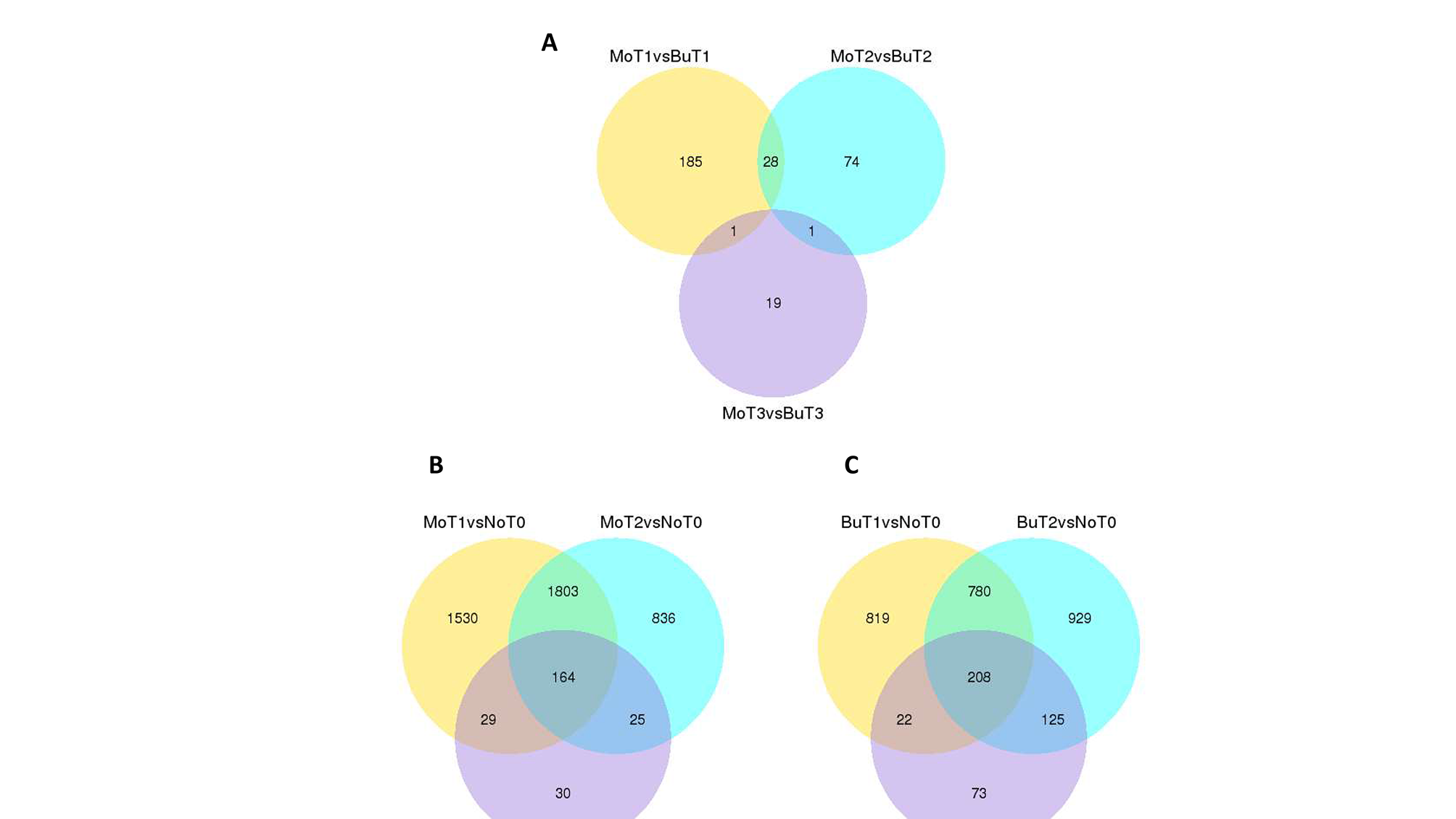

Supplement: Figure S6 — Venn diagrams of the groups. (A) Differentially expressed genes among the three groups of treated and control samples superimposed. (B,C), numbers of differentially expressed genes in the treated and control samples compared with the non-treated samples. NoT1, MoT1, BuT1, MoT2, BuT2, MoT3, BuT3 represent the 0-day non-treatment group, the 1-day treatment group, the 1-day control group, the 2-day treatment group, the 2-day control group, the 3-day treatment group, and the 3-day control group, respectively. [file Image6.TIF]

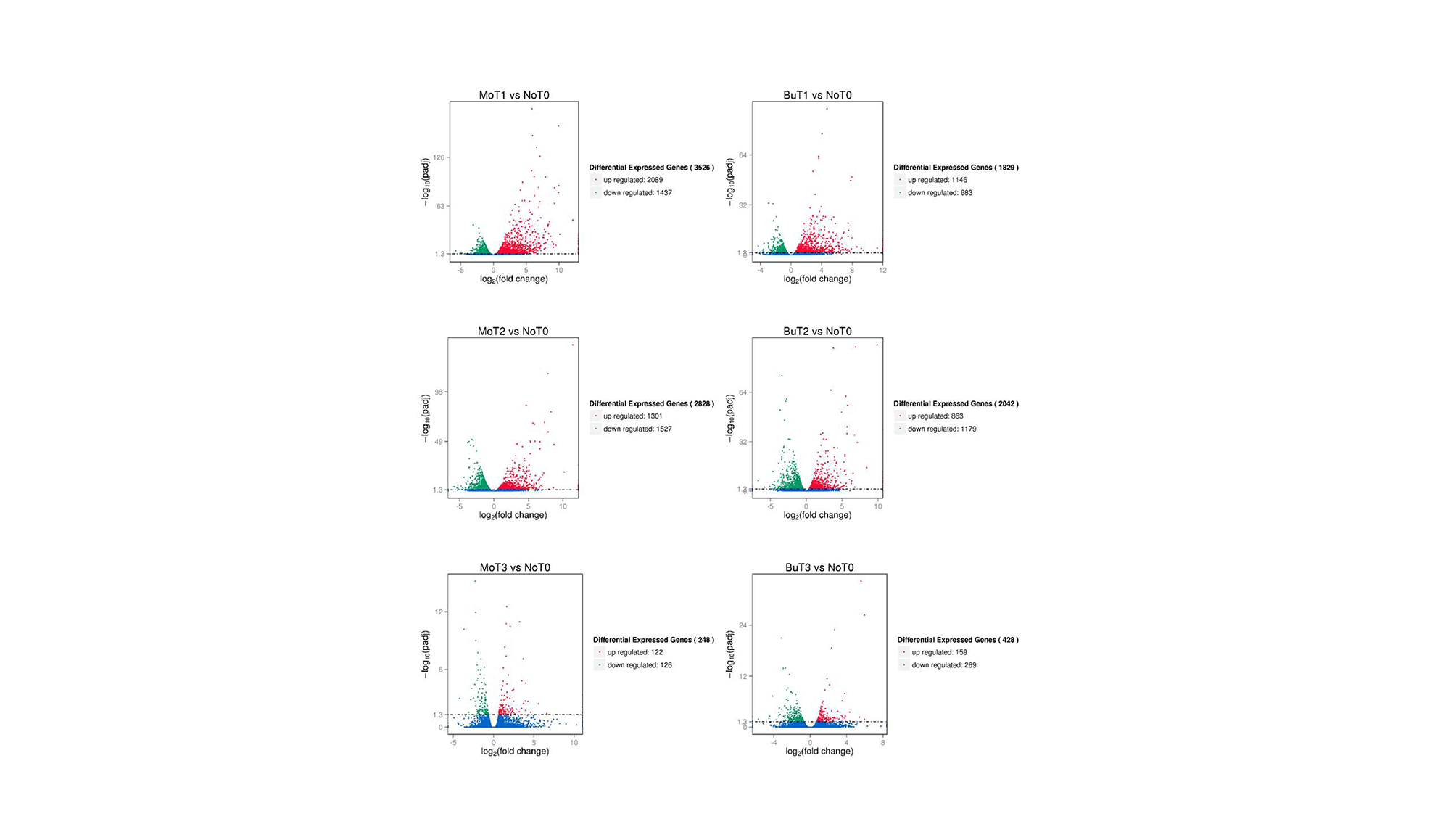

Supplement: Figure S7 — Volcano plots show that the treated rice samples have more differentially expressed genes relative to the non-treated rice samples than the control rice samples. MoT1 vs. NoT0, MoT2 vs. NoT0, and MoT3 vs. NoT0, and MoT3vsNoT0 represent the comparisons between the treated rice and the non-treated rice after MoHrip1 treatment for 1, 2, and 3 days. BuT1 vs. NoT0, BuT2 vs. NoT0, and BuT3 vs. NoT0 represent the comparisons between the control and non-treated rice groups after treatment with protein buffer for 1, 2, and 3 days. [file Image7.TIF]

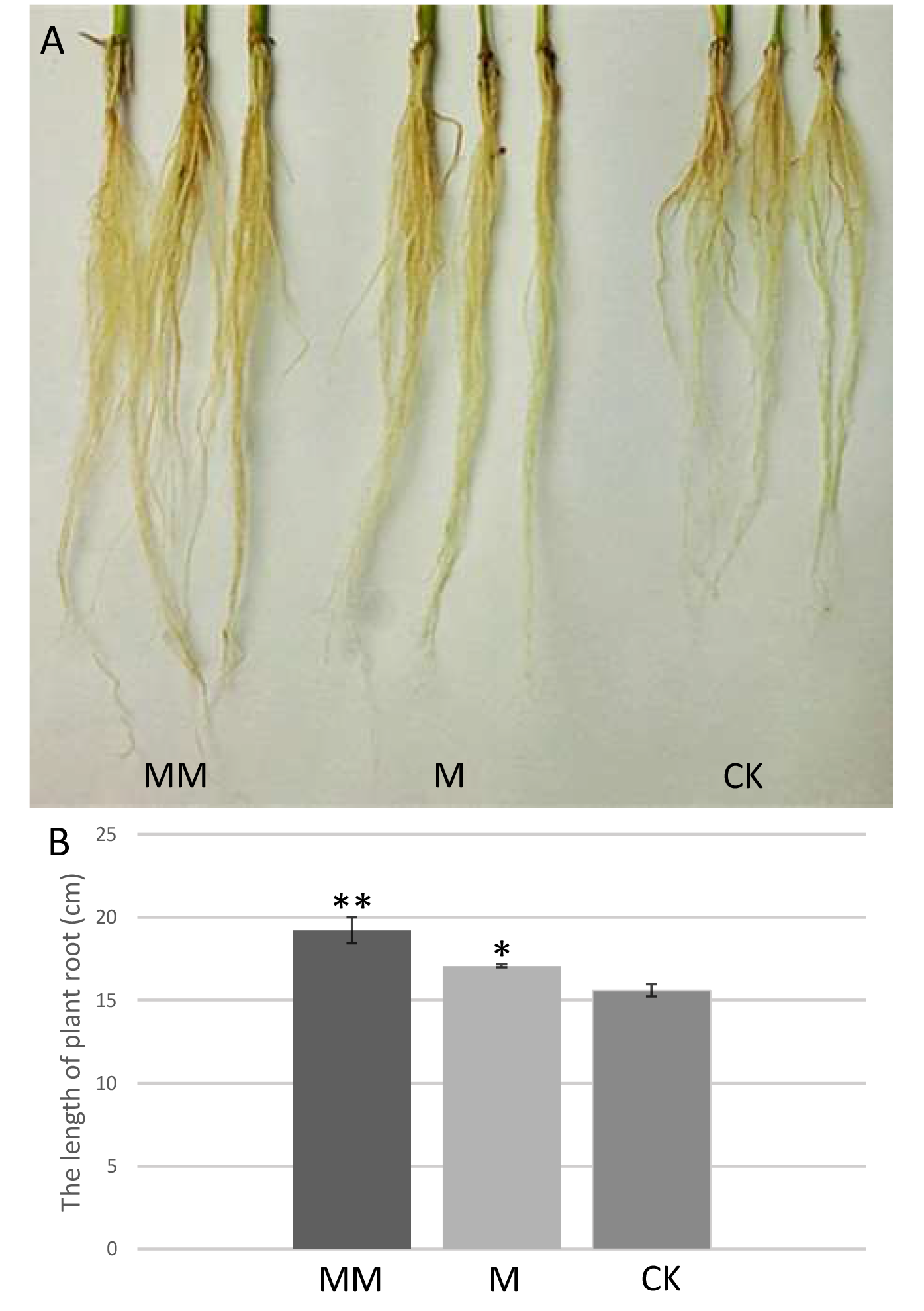

Supplement: Figure S8 — Root growth promotion of rice after treated with MoHrip1 or Tris-Cl. (A) Root growth promotion of rice plants. (B) Average length of rice plant root. MM (Rice immersed in MoHrip1 and sprayed with MoHrip1), M (Rice immersed in MoHrip1 and sprayed with Tris-Cl), and CK (Rice immersed in H2O and sprayed with Tris-Cl) were measured after 7 days inoculation with M. oryzae spores. Values are presented as the mean ± SD of three replicates, each consisting of 25 seedlings. Asterisks indicate statistically significant differences, as measured using SAS software (Duncan's t test, **p < 0.01, *p < 0.05). [file Image8.TIF]
