# Supplementary material for: Transcriptional Profiling of Rice Treated with MoHrip1 Reveal the Function of Protein Elicitor in Enhancement of Disease Resistance and Plant Growth
Source: Front Plant Sci. 2016 Dec 1;7:1818. doi: 10.3389/fpls.2016.01818 (PMC5131010; doi:10.3389/fpls.2016.01818)
Supplement: Table S1 — Primer sequences used in this study. [file Table1.DOCX]

| Table S1 Primer sequences used in this study | | | |
| --- | --- | --- | --- |
| Gene IDs | Gene name | Forwards primers | Reverse primers |
| OS11G0163100 | Osactin | GAGTATGATGAGTCGGGTCCAG | ACACCAACAATCCCAAACAGAG |
| OS01G0246700 | OsWRKY1 | GCATGAACGAGGAGAACCAG | CGGAGGCTGCATCATTTGAG |
| OS01G0826400 | OsWRKY24 | CCAAGAGATGGAGGAAAGAC | GATGTCGATGTCGCTCAT |
| OS05G0343400 | OsWRKY53 | CCAACACGGCAATACACACAT | TGGCCTGCAACGAATCAAC |
| OS05G0474800 | OsWRKY70 | TTACTCTTACACGAGCCAGCA | TGACGGCCCGATTAGATGAT |
| OS02G0181300 | OsWRKY71 | TGGATTAGCACCCAGCCTTC | AGGCTGCTGGTGAAAGAAGT |
| OS03G0180900 | OsJAZ2 | TTGCCTACCAGACATGCC | ACTCCGCCTCCTTCTTCT |
| OS03G0181100 | OsJAZ4 | TGACGACGCTGAGCCTGATGCC | AGACGACCATCCTCCCACCGTAG |
| OS04G0395800 | OsJAZ5 | ATGGCAACCGAGGCAACTAA | CTTGGAGAGGAATCGTTGCAG |
| OS02G0571100 | CPS2 | CTTGGAGTACGCCTACAG | GAACACCGATGATGAGACA |
| OS04G0178300 | CPS4 | TGGCGATGTATGGATTGG | TCCTCAAGTACCACCTTCTA |
| OS02G0569400 | CYP76M8 | ATGGAGAATAGCCAGATGTG | AAGTGTAGGTTGCCGATG |
| OS06G0569500 | KOL4 | GTGCACAGCTGACAGATGAC | TCGGATCTCTTGGTAGAGTAGC |
| OS02G0570400 | KSL7 | CCATATCCACAGCCAACA | ACTTAGTCCTCTCCTGATGT |
| OS12G0491800 | KSL10 | TAACCCTTGCCTCTGGGATG | ATTGTTCCACCGGAATCCCT |
| OS01G0816100 | NAC4 | AAGGCGCTCGTGTTCTACT | GCTTCTGTGAGCCCTTCTTG |
| OS03G0225900 | AOS2 | CCAAGAAAGAACGCCGAACA | CACCGTTCACGATGAACTCC |
| OS07G0677200 | POX22.3 | GCAATCCATCAAGTGTAT | CAGTTCATTAATCCTCCAT |
| OS07G0129200 | OsPR1a | GTCGGAGAAGCAGTGGTA | CGAGTAGTTGCAGGTGATG |
| OS12G0555200 | OsPR10b/PBZ1 | CTGTGGAAGGTCTGCTTGGA | TCTTGTATACGCTCCCTGCG |
| OS12G0555000 | OsPR10a | CTCAACCCTGCTGTGGAT | CTCGATCTTCGTCTCTGTCA |
| OS10G0542900 | OsPR3 | GTTCTGACCAGTTCCAGTG | GTAGTTCGATTGCCCTGTC |
| OS12G0628600 | OsPR5 | CAGCCAGGACTTCTACGA | TGTGTCTTGGTGTTGTCTTC |
| OS03G0764100 | ZFP1 | TCCGATCCGTCAGAGAGTTT | GGCTATGTATCACTCGCTTGC |
| OS01G0838600 | ZFP19 | ATCGGCGACGTTAGTGAT | GAACTCCTGCATGTCCCA |
| OS05G0427400 | PAL | GAGATCAACTCCGTCAAC | TGTAGAAGTCGTTCACCA |
