# Supplementary material for: Transcriptional Profiling of Rice Treated with MoHrip1 Reveal the Function of Protein Elicitor in Enhancement of Disease Resistance and Plant Growth
Source: Front Plant Sci. 2016 Dec 1;7:1818. doi: 10.3389/fpls.2016.01818 (PMC5131010; doi:10.3389/fpls.2016.01818)
Supplement: Table S2 — Statistics of the pathway enrichment of differentially expressed genes detected in treated rice after 1 day. A corrected p < 0.05 represents statistically significant enrichment of that pathway. [file Table2.DOCX]

| Table S2 Statistics of the pathway enrichment of differentially expressed genes detected in treated rice after 1 day. A corrected p-value of ＜0.05 represents statistically significant enrichment of that pathway. | | | | |
| --- | --- | --- | --- | --- |
| Term | Sample number | Bacgroud number | P-value | Corrected P-value |
| Phenylalanine, tyrosine and tryptophan biosynthesis | 7 | 42 | 4.41E-06 | 0.000154314 |
| Biosynthesis of secondary metabolites | 24 | 779 | 0.000233052 | 0.004078415 |
| Plant-pathogen interaction | 8 | 130 | 0.000642974 | 0.007261906 |
| Diterpenoid biosynthesis | 4 | 27 | 0.000829932 | 0.007261906 |
| Phenylpropanoid biosynthesis | 7 | 125 | 0.002344431 | 0.016411015 |
| Biosynthesis of amino acids | 9 | 207 | 0.003047382 | 0.017776397 |
