# Supplementary material for: Transcriptional Profiling of Rice Treated with MoHrip1 Reveal the Function of Protein Elicitor in Enhancement of Disease Resistance and Plant Growth
Source: Front Plant Sci. 2016 Dec 1;7:1818. doi: 10.3389/fpls.2016.01818 (PMC5131010; doi:10.3389/fpls.2016.01818)
Supplement: Table S3 — Ct and Log2 RPKM values for selected genes. Genes were designated as “undetected” when RPKM = 0.01. [file Table3.DOCX]

| Table S3. Ct and LOG2 RPKM values for selected genes. Genes were designated as ‘undetected’ when RPKM=0.01. | | | | | | | |
| --- | --- | --- | --- | --- | --- | --- | --- |
|  | OS01G0246700 | OS01G0826400 | OS05G0343400 | OS05G0474800 | OS02G0181300 | OS03G0180900 | OS04G0395800 |
| qRT-PCR | CT value | CT value | CT value | CT value | CT value | CT value | CT value |
| NoT1 | 30.02 | 31.43 | 27.62 | 34.85 | 28.18 | 30.26 | 33.22 |
| MoT1 | 26.75 | 28.43 | 25.53 | 28.61 | 25 | 26.11 | 29.43 |
| BuTI | 28.63 | 28.97 | 25.79 | 28.84 | 26.27 | 28.34 | 30.32 |
| MoT2 | 28.51 | 30.79 | 25.87 | 30.63 | 27.06 | 28.76 | 30.4 |
| BuT2 | 29.77 | 30.2 | 25.79 | 31.8 | 27.86 | 29.17 | 31.08 |
| MoT3 | 29.22 | 31.3 | 25.4 | 30.9 | 27.08 | 28.83 | 30.34 |
| BuT3 | 30.14 | 32.69 | 25.37 | 31.12 | 27.47 | 30.4 | 31.06 |
|  |  |  |  |  |  |  |  |
|  | OS01G0246700 | OS01G0826400 | OS05G0343400 | OS05G0474800 | OS02G0181300 | OS03G0180900 | OS04G0395800 |
| RNA-seq | LOG2 RPKM | LOG2 RPKM | LOG2 RPKM | LOG2 RPKM | LOG2 RPKM | LOG2 RPKM | LOG2 RPKM |
| NoT1 | 0.430159192 | -0.229488164 | 4.208308595 | -4.513198272 | 3.134313861 | -0.260389367 | -2.63684324 |
| MoT1 | 4.925211335 | 4.017244164 | 6.409550914 | 2.836251518 | 6.039202675 | 5.670915578 | 3.429753205 |
| BuTI | 3.778604936 | 2.985509172 | 5.623061369 | 1.63778654 | 5.016605785 | 3.928147081 | 1.683135911 |
| MoT2 | 3.210781695 | 1.577863289 | 5.135740449 | 0.478579403 | 4.046870648 | 2.980926603 | 1.085544717 |
| BuT2 | 2.378936825 | 1.054251281 | 4.72197851 | -0.169158857 | 3.727437343 | 1.080928193 | -1.33199031 |
| MoT3 | 2.35745948 | 1.895823079 | 5.253568143 | 0.393835213 | 4.25309396 | 2.172347995 | 0.286449235 |
| BuT3 | 1.465297725 | 0.033175636 | 4.664619203 | -1.355700145 | 3.457022575 | 1.081209537 | -1.12125925 |
|  |  |  |  |  |  |  |  |
|  |  |  |  |  |  |  |  |
|  | OS06G0569500 | OS02G0570400 | OS12G0491800 | OS01G0816100 | OS07G0677200 | OS07G0129200 | OS12G0555200 |
| qRT-PCR | CT value | CT value | CT value | CT value | CT value | CT value | CT value |
| NoT1 | 30.76 | 33.72 | 32.83 | 32.31 | 27.01 | 31.15 | 32.17 |
| MoT1 | 25.01 | 27.98 | 27.64 | 28.24 | 23.4 | 23.96 | 26.43 |
| BuTI | 26.36 | 28.87 | 28.37 | 30.92 | 24.08 | 24.39 | 26.37 |
| MoT2 | 27.04 | 28.37 | 29.54 | 30.13 | 23.23 | 24 | 26.66 |
| BuT2 | 29.66 | 31.41 | 30.57 | 32.27 | 23.59 | 25.53 | 27.15 |
| MoT3 | 29.38 | 30.73 | 30.88 | 31.65 | 24.28 | 23.34 | 27.84 |
| BuT3 | 30.52 | 32.84 | 34.71 | 32.42 | 24.34 | 31.16 | 28.77 |
|  |  |  |  |  |  |  |  |
|  | OS06G0569500 | OS02G0570400 | OS12G0491800 | OS01G0816100 | OS07G0677200 | OS07G0129200 | OS12G0555200 |
| RNA-seq | LOG2 RPKM | LOG2 RPKM | LOG2 RPKM | LOG2 RPKM | LOG2 RPKM | LOG2 RPKM | LOG2 RPKM |
| NoT1 | 1.766325465 | -6.64385619 | -2.651056527 | -1.693764986 | 1.877526127 | -6.64385619 | -2.43430064 |
| MoT1 | 6.578713393 | 4.823936738 | 4.558656599 | 3.593764098 | 8.216853722 | 3.878848739 | 7.791953075 |
| BuTI | 4.952577183 | 3.146561364 | 3.144002514 | 1.81912586 | 6.846627481 | 2.27683536 | 6.437176988 |
| MoT2 | 4.619626858 | 3.422397528 | 2.722655515 | 1.610400696 | 7.676417566 | 3.840674625 | 6.321857167 |
| BuT2 | 2.375004897 | 0.889427162 | -0.117572763 | 0.049590999 | 6.057338179 | 1.246234663 | 4.541704853 |
| MoT3 | 2.892426669 | 1.467441307 | 0.81081931 | 0.516915357 | 5.76857954 | 2.4996522 | 4.927749242 |
| BuT3 | 1.647792915 | -1.612349719 | -3.043980971 | 0.187553397 | 3.754397256 | -2.256244601 | 2.861537569 |
|  |  |  |  |  |  |  |  |
|  |  |  |  |  |  |  |  |
|  | OS12G0555000 | OS10G0542900 | OS12G0628600 | OS03G0764100 | OS05G0427400 |  |  |
| qRT-PCR | CT value | CT value | CT value | CT value | CT value |  |  |
| NoT1 | 30.01 | 31.57 | 32.56 | 23.27 | 31.95 |  |  |
| MoT1 | 22.6 | 25.72 | 20.59 | 24.93 | 29.32 |  |  |
| BuTI | 23.06 | 26.56 | 22.02 | 24.8 | 31.02 |  |  |
| MoT2 | 21.23 | 23.63 | 20.7 | 24.15 | 31.42 |  |  |
| BuT2 | 22.36 | 26.53 | 21.08 | 24 | 32.56 |  |  |
| MoT3 | 21.02 | 26.37 | 22.8 | 23.78 | 31.55 |  |  |
| BuT3 | 22.95 | 27.07 | 23.09 | 24.22 | 33.4 |  |  |
|  |  |  |  |  |  |  |  |
|  | OS12G0555000 | OS10G0542900 | OS12G0628600 | OS03G0764100 | OS05G0427400 |  |  |
| RNA-seq | LOG2 RPKM | LOG2 RPKM | LOG2 RPKM | LOG2 RPKM | LOG2 RPKM |  |  |
| NoT1 | -0.773382555 | -1.872391922 | -3.943943052 | 5.460248804 | 1.605936958 |  |  |
| MoT1 | 9.541796706 | 6.311756805 | 8.48979082 | 5.63286959 | 4.434564481 |  |  |
| BuTI | 8.68471985 | 5.20691264 | 7.574401615 | 4.62068905 | 2.721969628 |  |  |
| MoT2 | 7.552494941 | 6.531142569 | 7.948674236 | 4.989770596 | 1.988054977 |  |  |
| BuT2 | 6.375914018 | 4.799176134 | 6.180345928 | 3.758898849 | 0.577326941 |  |  |
| MoT3 | 6.953184087 | 4.838651491 | 6.636024701 | 5.260461589 | 1.371218428 |  |  |
| BuT3 | 5.380731289 | 2.599175227 | 4.048099712 | 3.543102104 | 0.261466584 |  |  |
