# Supplementary material for: Transcriptional Profiling of Rice Treated with MoHrip1 Reveal the Function of Protein Elicitor in Enhancement of Disease Resistance and Plant Growth
Source: Front Plant Sci. 2016 Dec 1;7:1818. doi: 10.3389/fpls.2016.01818 (PMC5131010; doi:10.3389/fpls.2016.01818)
Supplement: Table S4 — List of the gene IDs and their functional descriptions from two detailed gene clustering analyses. [file Table4.DOCX]

| Table S4. List of the gene IDs and their functional descriptions from two detailed gene clustering analyses. | |
| --- | --- |
| geneID | Description |
| OS03G0168200 | Protein of unknown function DUF3110 |
| OS04G0596300 | Protein of unknown function DUF760 |
| OS03G0235000 | prx38, prxRPN |
| OS01G0597800 | UDP-glucuronosyl/UDP-glucosyltransferase family protein. |
| OS06G0354700 | Alpha/beta hydrolase-fold family protein |
| OS01G0342750 | Plant PDR ABC transporter |
| OS07G0218700 | Cytochrome P450, E-class |
| OS04G0172400 | Transferase family protein,OsAT19 |
| OS03G0126000 | Phosphorybosyl anthranilate transferase 1(PAT) |
| OS07G0182100 | Tryptophan synthase alpha chain (TS) |
| OS05G0530400 | Heat stress transcription factor Spl7 |
| OS06G0323100 | SAM dependent carboxyl methyltransferase |
|  |  |
|  |  |
| geneID | Description |
| OS05G0197300 | IQ calmodulin-binding region domain containing protein |
| OS09G0332700 | PDR-type ABC transporter,PDR20 |
| OS07G0262200 | Prohibitin |
| OS08G0485400 | Oxidoreductase,2-nitropropane dioxygenase |
| OS10G0527800 | Tau class GST protein, OsGSTU12 |
| OS02G0274100 | Peroxisomal fatty acid beta-oxidation multifunctional protein (MFP) |
| OS08G0320400 | IGPS(Indole-3-Glycerol Phosphate Synthase ) |
| OS06G0133900 | EPSPS |
| OS01G0375200 | DHQDT/SDH |
| OS05G0115100 | Protein of unknown function DUF1635 |
| OS06G0650900 | Conserved hypothetical protein |
| OS07G0586100 | Early nodule-specific protein |
