# Supplementary material for: Transcriptional Profiling of Rice Treated with MoHrip1 Reveal the Function of Protein Elicitor in Enhancement of Disease Resistance and Plant Growth
Source: Front Plant Sci. 2016 Dec 1;7:1818. doi: 10.3389/fpls.2016.01818 (PMC5131010; doi:10.3389/fpls.2016.01818)
Supplement: Table S5 — Eighty genes with particular expression patterns in the treated rice and low or undetected expression in the control rice. Gene IDs with bold fonts were selected for qRT-PCR validation. [file Table5.DOCX]

| Table S5. Eighty genes with particular expression patterns in the treated rice and low or undetected expression in the control rice. Gene IDs with bold fonts were selected for qRT-PCR validation. | |
| --- | --- |
| geneID | Description |
| OS03G0187550 | Hypothetical gene |
| **OS01G0694100** | Leucine-rich repeat domain containing protein |
| **OS12G0215950** | Leucine Rich Repeat family protein, expressed |
| OS07G0518100 | Cytochrome P450 family protein |
| OS02G0548700 | ubiquitin-protein ligase |
| **OS02G0733500** | EF-Hand type domain containing |
| OS06G0529900 | Hypothetical conserved gene |
| OS08G0206400 | Similar to Potassium transporter 18 |
| OS03G0326200 | Similar to aminophospholipid ATPase |
| **OS01G0787600** | Salicylic acid-binding protein 2 |
| OS06G0282000 | UDP-glucuronosyl/UDP-glucosyltransferase family protein |
| OS01G0734000 | Similar to WRKY DNA binding protein（WRKY23） |
| OS03G0389601 | Hypothetical protein |
| OS08G0524400 | Protein of unknown function DUF568, DOMON-like domain containing protein. |
| OS11G0687200 | von Willebrand factor, type A domain containing protein. |
| OS11G0130400 | Harpin-induced 1 domain containing protein. |
| OS01G0160100 | Similar to Pyruvate decarboxylase isozyme 2 |
| OS07G0117400 | NB-ARC domain containing |
| OS03G0829200 | Similar to epoxide hydrolase 2 |
| OS01G0629600 | Peptidase S10, serine carboxypeptidase family protein |
| OS03G0770800 | CSLC9 |
| OS02G0621400 | Hypothetical protein |
| OS04G0172400 | Transferase family protein(OsAT19) |
| OS03G0808350 | Hypothetical protein |
| OS10G0575000 | Transcription factor MYC7E(OsbHLH009, OsMYC2) |
| OS06G0216300 | 12-oxophytodienoic acid reductase（OsOPR1, OsOPR2, OsOPR11） |
| **OS08G0398300** | ABC transporter-like domain containing protein.（OsABCA4） |
| OS08G0526100 | NAD(P)-binding domain containing protein(OsUGlcAE2) |
| OS05G0542150 | Conserved hypothetical protein |
| OS02G0650900 | Similar to Glutamate dehydrogenase 2(OsGDH3) |
| OS12G0624800 | Conserved hypothetical protein |
| OS01G0233900 | Ubiquitin-conjugating enzyme 37 |
| OS01G0958900 | OsMLH1 |
| OS10G0410900 | Hypothetical protein |
| OS03G0177966 | Hypothetical protein |
| OS12G0477050 | Conserved hypothetical protein |
| OS09G0471900 | Bacterial Fmu (Sun)/eukaryotic nucleolar NOL1/Nop2p domain containing protein |
| OS10G0578700 | Hypothetical protein |
| OS04G0219700 | Conserved hypothetical protein |
| OS04G0433800 | ATP binding / ATP-dependent helicase/ helicase/ nucleic acid binding(OsRecQl4) |
| OS11G0110000 | Conserved hypothetical protein |
| OS02G0687550 | Hypothetical gene |
| **OS01G0158900** | Zinc finger, NF-X1-type domain containing protein |
| OS07G0194100 | Similar to OSK2 (Fragment) |
| OS04G0448100 | Protein of unknown function DUF1644 family protein. |
| OS07G0229100 | Cyclin-like F-box domain containing protein.(OsFbox354) |
| OS11G0547000 | Similar to FKF1(OsFbox609, Os_F0370) |
| OS04G0571200 | Similar to OSIGBa0111L12.9 protein,OsRFPH2-14 |
| OS09G0549400 | Hypothetical protein |
| OS08G0504800 | Conserved hypothetical protein |
| OS06G0546400 | Similar to 50S ribosomal protein L1 |
| OS08G0404350 | Similar to FACT complex subunit SPT16 |
| **OS11G0578100** | Heat shock protein DnaJ, N-terminal domain containing protein.OsDjC76 |
| OS09G0509500 | Diacylglycerol acyltransferase domain containing protein. |
| OS06G0690900 | Pentatricopeptide repeat domain containing protein |
| OS12G0638800 | Similar to HI0933-like protein, expressed |
| OS05G0241100 | Hypothetical protein |
| OS07G0530100 | Tyrosyl-DNA phosphodiesterase family protein |
| OS08G0453700 | OsNox6、Similar to ATRBOH F (ARABIDOPSIS THALIANA RESPIRATORY BURST OXIDASE PROTEIN F); NAD(P)H oxidase. |
| OS06G0298000 | Conserved hypothetical protein |
| OS05G0295900 | Conserved hypothetical protein |
| OS02G0801200 | Similar to Cyclin B |
| OS09G0475700 | Peptidase S9A, prolyl oligopeptidase family protein |
| OS03G0396900 | Similar to PIMT |
| OS03G0110800 | OsDRM2, Similar to DNA methyltransferase |
| OS01G0788900 | Pentatricopeptide repeat domain containing protein |
| OS10G0495200 | Similar to Fertility restorer |
| OS11G0104833 | Hypothetical protein |
| OS12G0442800 | Similar to Sulfite oxidase |
| OS07G0122300 | Protein of unknown function DUF1719 |
| **OS01G0601625** | Leucine rich repeat, N-terminal domain containing protein |
| OS03G0179000 | Similar to Tubulin-tyrosine ligase family protein, expressed. |
| OS09G0442900 | Similar to Axi 1 (Auxin-independent growth promoter)-like protein |
| OS02G0122100 | Similar to JHL05D22.13 protein. |
| OS07G0603100 | Nucleotide-binding, alpha-beta plait domain containing protein |
| OS03G0198200 | Conserved hypothetical protein |
| **OS05G0179000** | Zinc finger, RING/FYVE/PHD-type domain containing protein |
| OS11G0655000 | Hypothetical protein |
| OS07G0529600 | OsDR8, Similar to Thiazole biosynthetic enzyme 1-1, chloroplast precursor. |
| OS12G0596600 | Conserved hypothetical protein |
